# Pioneering perovskite quantum dot nanosensors for heavy metal ion detection: mechanisms, design, and industrial applications

**DOI:** 10.1039/d5ra06200d

**Published:** 2025-10-23

**Authors:** Suleiman Ibrahim Mohammad, Asokan Vasudevan, I. B. Sapaev, Munthar Kadhim Abosaoda, Chou-Yi Hsu, Malatesh Akkur, Alok Kumar Mishra, Gaganjot Kaur, Rajesh Singh, Ahmad Mohebi

**Affiliations:** a Electronic Marketing and Social Media, Economic and Administrative Sciences, Zarqa University Jordan; b Research Follower, INTI International University 71800 Negeri Sembilan Malaysia; c Faculty of Business and Communications, INTI International University 71800 Negeri Sembilan Malaysia; d Shinawatra University 99 Moo 10, Bangtoey, Samkhok Pathum Thani 12160 Thailand; e Head of the Department Physics and Chemistry, “Tashkent Institute of Irrigation and Agricultural Mechanization Engineers” National Research University Tashkent Uzbekistan; f Scientific Researcher of the University of Tashkent for Applied Science Uzbekistan; g School of Engineering, Central Asian University Tashkent 111221 Uzbekistan; h Western Caspian University, Scientific Researcher Baku Azerbaijan; i College of Pharmacy, The Islamic University Najaf Iraq; j Department of Medical Analysis, Medical Laboratory Technique College, The Islamic University of Al Diwaniyah Al Diwaniyah Iraq; k Department of Pharmacy, Chia Nan University of Pharmacy and Science Tainan 71710 Taiwan; l Department of Physics & Electronics, School of Sciences, JAIN (Deemed to be University) Bangalore Karnataka India; m Department of Electrical & Electronics Engineering, Siksha 'O' Anusandhan (Deemed to be University) Bhubaneswar Odisha 751030 India; n Department of Electronics and Communication Engineering, Chandigarh University Mohali Punjab India; o Uttaranchal Institute of Technology, Uttaranchal University Dehradun 248007 Uttarakhand India; p Department of Chemistry, Young Researchers and Elite Club, Islamic Azad University Tehran Branch Tehran Iran a.mohebiacademic@gmail.com

## Abstract

Perovskite quantum dots (PQDs) are a transformative platform for ultrasensitive heavy metal ion detection, leveraging high photoluminescence quantum yield (PLQY, 50–90%), narrow emission spectra (FWHM 12–40 nm), tunable bandgaps, and defect-tolerant optoelectronic properties. This review is the first systematic evaluation of PQD-based nanosensors for detecting Hg^2+^, Cu^2+^, Cd^2+^, Fe^3+^, Cr^6+^, and Pb^2+^, elucidating key mechanisms like cation exchange, electron/hole transfer, Förster resonance energy transfer, and surface trap-mediated quenching. Advanced synthesis methods, including hot-injection, ligand-assisted reprecipitation, and microwave-assisted techniques, enable precise control over size, crystallinity, and surface chemistry. Lead-based PQDs (*e.g.*, CsPbX_3_) achieve limits of detection (LODs) as low as 0.1 nM with rapid response times (<10 s), while lead-free variants (*e.g.*, Cs_3_Bi_2_X_9_, CsSnX_3_) offer eco-friendly alternatives with enhanced aqueous stability. PQD@MOF composites and ratiometric designs enhance selectivity in complex matrices, surpassing carbon quantum dots and semiconductor QDs in sensitivity and versatility. Applications include industrial wastewater remediation, lubricant quality control, and environmental compliance, ensuring ecosystem protection and product integrity. This seminal work addresses challenges like aqueous instability, Pb toxicity, scalability, and matrix interference, benchmarking PQDs against alternative nanomaterials. Future directions include comparisons with other nanoparticles, multiplexed sensing platforms, and sustainable lead-free innovations. By integrating fundamental insights with practical applications, this review establishes PQDs as a high-impact paradigm for advancing heavy metal ion sensing in industrial and environmental contexts, guiding innovations in sensitivity, selectivity, and scalability.

## Abstract 2

Review pioneers perovskite quantum dots for heavy metal detection, achieving sub-nanomolar sensitivity. Lead-free variants enable eco-friendly wastewater and lubricant monitoring.

## Graphical abstract

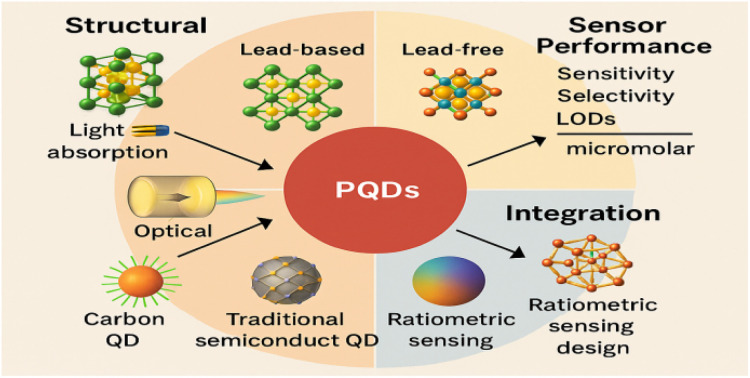

## 1. Introduction

Heavy metal ion contamination in environmental, industrial, and biological systems poses significant risks to ecosystems, human health, and industrial processes due to their toxicity, persistence, and bioaccumulation.^1–3^ Elements such as mercury (Hg^2+^), copper (Cu^2+^), cadmium (Cd^2+^), iron (Fe^3+^), chromium (Cr^6+^), and lead (Pb^2+^) are prevalent pollutants in industrial wastewater, lubricants, and natural water bodies, necessitating robust detection methods to ensure environmental compliance and product integrity.^4–6^ Traditional sensing technologies, including atomic absorption spectroscopy,^7,8^ inductively coupled plasma mass spectrometry,^9,10^ and electrochemical methods,^11,12^ offer high sensitivity but are often hindered by high costs, complex instrumentation, and limited portability, making them less suitable for real-time or on-site applications.^13,14^ In response, nanomaterial-based nanosensors have emerged as promising alternatives, offering rapid response, portability, and cost-effectiveness.^15^ Among these, perovskite quantum dots (PQDs) have garnered significant attention due to their exceptional optoelectronic properties, positioning them as a transformative platform for heavy metal ion detection.^16,17^

PQDs, characterized by the general formula ABX_3_ (where A is a monovalent cation, B is a divalent metal cation, and X is a halide anion), exhibit unique attributes that make them ideal for sensing applications.^18,19^ Their high photoluminescence quantum yield (PLQY, 50–90%), narrow emission spectra (full width at half maximum (FWHM) 12–40 nm), and tunable bandgaps enable ultrasensitive detection with high color purity and specificity. These properties arise from quantum confinement effects at nanoscale dimensions (2–10 nm), which result in discrete energy levels and enhanced oscillator strengths.^20,21^ Additionally, PQDs possess large absorption coefficients (10^5^ to 10^6^ cm^−1^) and fast radiative recombination rates, facilitating efficient light harvesting and stable fluorescence signals critical for detecting analyte-induced changes. The structural versatility of PQDs allows compositional tuning through substitutions at the A, B, or X sites, enabling tailored optical and electronic properties for specific sensing needs.^19,20^ Lead-based PQDs, such as CsPbX_3_ and CH_3_NH_3_PbX_3_, are renowned for their superior PLQY and tunable emission across the visible spectrum, while lead-free variants, such as Cs_3_Bi_2_X_9_ and CsSnX_3_, address toxicity concerns, offering eco-friendly alternatives with improved stability in aqueous environments.^22^

The detection of heavy metal ions using PQDs relies on their ability to undergo fluorescence quenching or enhancement triggered by interactions with analytes. Mechanisms such as cation exchange, electron transfer, and Förster Resonance Energy Transfer (FRET) underpin these responses, driven by the high surface-to-volume ratio and defect-tolerant nature of PQDs.^23,24^ For instance, the ionic radius similarity between Pb^2+^ and Hg^2+^ facilitates rapid cation exchange in lead-based PQDs, leading to efficient quenching, while electron transfer dominates in interactions with transition metal ions like Cu^2+^ or Fe^3+^.^17,21^ Surface ligands, such as oleylamine or poly(ethylenimine) (PEI), play a critical role in modulating selectivity and sensitivity by passivating defects and mediating ion–PQD interactions. Advanced synthesis methods, including hot-injection, ligand-assisted reprecipitation, and microwave-assisted techniques, enables precise control over PQD size, crystallinity, and surface chemistry, directly impacting their sensing performance.^25^

The industrial relevance of PQD-based nanosensors lies in their ability to address critical monitoring needs in sectors such as chemical processing, wastewater treatment, and lubricant quality control.^26,27^ Their high sensitivity and rapid response times make them suitable for detecting trace contaminants in real-time, ensuring compliance with stringent environmental regulations and maintaining product quality. However, challenges such as the aqueous instability of lead-based PQDs, toxicity concerns, and scalability of synthesis methods must be addressed to facilitate widespread adoption. Emerging strategies, including the development of lead-free PQDs, advanced encapsulation techniques, and computational modeling, offer pathways to overcome these limitations, paving the way for practical implementation.^28,29^

**Fig. 1:**
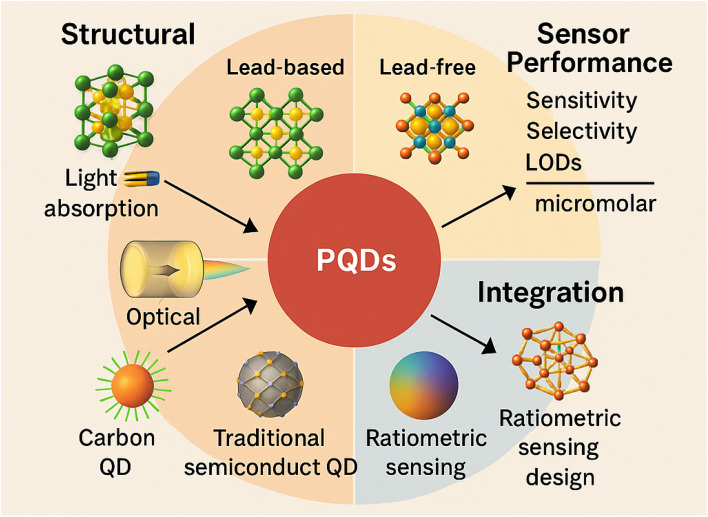
Overview of PQD-based nanosensors for heavy metal ion detection, highlighting structure, performance, integration strategies, and comparison with other quantum dots.

This review provides the first comprehensive analysis of PQD-based nanosensors for heavy metal ion detection, systematically exploring their structural, optical, and mechanistic foundations. It covers diverse PQD types, including lead-based (*e.g.*, CsPbBr_3_) and lead-free (*e.g.*, Cs_3_Bi_2_Br_9_) systems, their synthesis methods, and their advantages and limitations for sensing applications. The review evaluates sensor performance, focusing on sensitivity, selectivity, and limits of detection (LODs) ranging from sub-nanomolar (*e.g.*, 0.1 nM for Cu^2+^ in organic solvents) to micromolar levels, depending on the ion and matrix. Integration with stable matrices like metal–organic frameworks and ratiometric sensing designs enhances performance in complex industrial and environmental matrices. By comparing PQDs with other nanomaterials, such as carbon QDs and traditional semiconductor quantum dots, and addressing both fundamental and applied aspects, this work aims to guide future research, fostering innovations to improve the sensitivity, selectivity, and scalability of PQD-based nanosensors for diverse heavy metal ion detection applications (Fig. 1).

## 2. Fundamentals of perovskite quantum dots

### 2.1. Structural and optical properties of perovskite quantum dots

PQDs are nanoscale semiconductor materials characterized by the general formula ABX_3_, where A is a monovalent cation (*e.g.*, Cs^+^, methylammonium (MA^+^), or formamidinium (FA^+^)), B is a divalent metal cation (*e.g.*, Pb^2+^, Sn^2+^, or bismuth (Bi^3+^)), and X is a halide anion (*e.g.*, Cl^−^, Br^−^, or I^−^). The crystal structure of PQDs typically adopts a cubic, tetragonal, or orthorhombic lattice in 3D ABX_3_ forms, depending on the composition and temperature.^30,31^ In this 3D structure, the B cation is coordinated octahedrally by six X anions, forming interconnected [BX_6_]^4−^ octahedra that create a three-dimensional framework, with A cations occupying cuboctahedral voids for charge neutrality. This extended connectivity enables efficient charge transport and high PLQY (50–90%). In contrast, 0D/2D derivatives like A_3_B_2_X_9_ (*e.g.*, Cs_3_Bi_2_X_9_) feature isolated [B_2_X_9_]^3−^ dimers or layered structures with reduced octahedral connectivity, resulting in stronger quantum confinement, broader emission spectra (FWHM ∼40–60 nm), and typically lower PLQY (20–50%), which impacts sensitivity in sensing applications due to increased defect states.^19,28,40^ The structural versatility of perovskites enables compositional tuning at A, B, or X sites to modulate properties critical for heavy metal ion detection.

The stability of the perovskite lattice is governed by the Goldschmidt tolerance factor (*t*), defined as:1
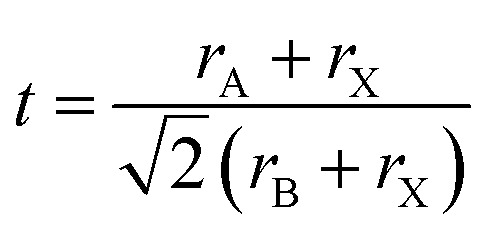
where *r*_A_, *r*_B_, and *r*_X_ are the ionic radii of A, B, and X ions, respectively. A tolerance factor between 0.8 and 1.0 ensures a stable 3D cubic structure, while lower values favor 0D/2D phases. For instance, CsPbBr_3_ (*t* ≈ 0.81) adopts a cubic 3D structure at room temperature, offering high optical efficiency, whereas Cs_3_Bi_2_Br_9_ (*t* < 0.8) forms a 0D layered structure, enhancing aqueous stability but reducing radiative efficiency.^32,33,43^

The optical properties of PQDs are dominated by quantum confinement effects due to their nanoscale dimensions (2–10 nm), resulting in discrete energy levels and size-dependent bandgaps. This confinement enhances oscillator strength, leading to high PLQY (50–90% for 3D ABX_3_; 20–50% for 0D/2D A_3_B_2_X_9_) and narrow emission spectra (FWHM 12–40 nm for 3D; broader for 0D/2D). For example, 3D CsPbBr_3_ emits green light at ∼520 nm with FWHM ∼20 nm, ideal for selective sensing, while 0D Cs_3_Bi_2_Br_9_ shows blue emission (∼450 nm) with higher stability in polar media.^15,26,43^ The bandgap can be tuned by size or halide composition; smaller PQDs or Cl-rich 3D variants exhibit wider bandgaps (∼3.0 eV for CsPbCl_3_), while I-rich 3D forms have narrower ones (∼1.7 eV for CsPbI_3_). 0D/2D structures often display wider effective bandgaps due to confinement. PQDs possess large absorption coefficients (10^5^ to 10^6^ cm^−1^) and fast radiative recombination rates, with defect tolerance minimizing non-radiative losses—more pronounced in 3D forms where defects lie within bands.^30,34^

The surface chemistry of PQDs significantly influences their properties. PQDs are capped with ligands like oleylamine (OAm) or oleic acid (OA), which passivate defects and enhance stability. In 3D ABX_3_, ligands primarily provide steric protection, while in 0D/2D A_3_B_2_X_9_, they also mitigate interlayer defects for better aqueous sensing.^18,35^ Exciton dynamics in PQDs feature short radiative lifetimes (1–10 ns) and high binding energies (20–100 meV), enabling sensitive energy transfer for detection. The high surface-to-volume ratio amplifies ion interactions, with 0D/2D structures showing enhanced trap states for quenching but potentially slower kinetics compared to 3D forms.^14,36^

#### 2.1.1. Crystal structure and compositional flexibility

A tolerance factor between 0.8 and 1.0 typically ensures a stable cubic perovskite structure, while deviations may lead to tetragonal or orthorhombic phases, impacting optical and sensing performance. For instance, CsPbBr_3_ adopts a cubic structure at room temperature, offering high symmetry and optical efficiency, whereas CH_3_NH_3_PbBr_3_ may transition to a tetragonal phase under certain conditions, affecting its photoluminescence stability.^32,33^ The ability to substitute halides (*e.g.*, Cl^−^ for Br^−^) or cations (*e.g.*, Sn^2+^ for Pb^2+^) allows precise control over the lattice parameters, enabling tailored bandgap energies and emission properties.

#### 2.1.2. Quantum confinement and optical properties

The optical properties of PQDs are dominated by quantum confinement effects due to their nanoscale dimensions (2–10 nm), which result in discrete energy levels and size-dependent bandgaps. This confinement enhances the oscillator strength, leading to high photoluminescence quantum yields (PLQY), often exceeding 50–90%, and narrow emission spectra with full width at half maximum (FWHM) values of 12–40 nm. For example, CsPbBr_3_ PQDs emit green light at ∼520 nm with a FWHM of ∼20 nm, ensuring high color purity ideal for sensing applications.^15,26^ The bandgap can be tuned by adjusting the particle size or halide composition; smaller PQDs or those with higher Cl content exhibit wider bandgaps (*e.g.*, ∼3.0 eV for CsPbCl_3_), while larger PQDs or I-rich compositions have narrower bandgaps (*e.g.*, ∼1.7 eV for CsPbI_3_). PQDs possess large absorption coefficients (10^5^ to 10^6^ cm^−1^), enabling efficient light harvesting, and fast radiative recombination rates due to their direct bandgap nature. Their high defect tolerance, where defect states often lie within the conduction or valence bands rather than the bandgap, minimizes non-radiative recombination, enhancing PL efficiency.^30,34^ This property is critical for sensing, as it ensures strong and stable fluorescence signals responsive to analyte-induced changes, such as quenching or enhancement.

**Fig. 2:**
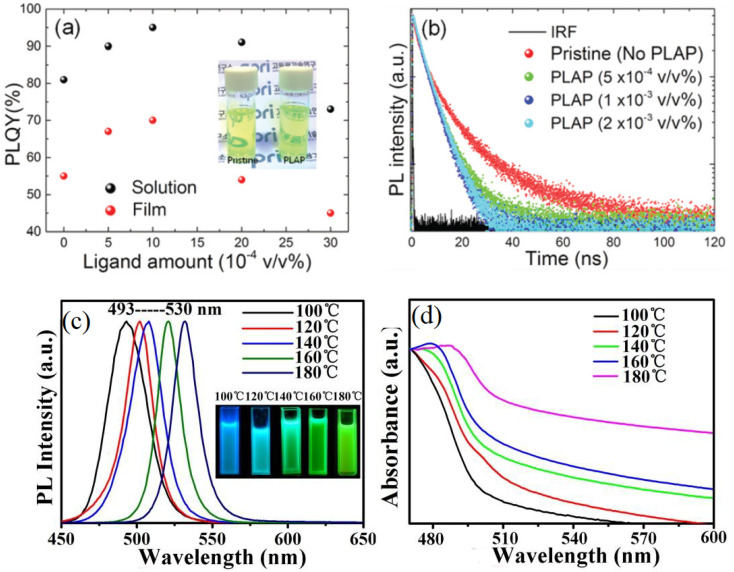
(a) PLQY of PQDs as a function of ligand amount in solution and film, (b) time-resolved PL decay curves for pristine PQDs and PLAP-treated PQDs at different concentrations. Reprinted with permission from ref. 20. Copyright 2018, Royal Society of Chemistry. (c) PL spectra of PQDs at various temperatures, and (d) absorbance spectra of PQDs at different temperatures. Adapted with permission from ref. 21. Copyright 2017, Elsevier.

Panel (a) demonstrates the PLQY of PQDs as a function of ligand amount (10^4^ v/v%), comparing solution and film states, with PLQY values reaching up to 90%, reflecting the high oscillator strength and quantum confinement effects due to their nanoscale dimensions (2–10 nm).^20^ This high PLQY, often exceeding 50–90%, is attributed to the discrete energy levels and size-dependent bandgaps, enhancing color purity with narrow emission spectra (FWHM 12–40 nm). Panel (b) presents time-resolved PL decay curves for pristine PQDs and those treated with PLAP at concentrations of 5 × 10^3^, 1 × 10^3^, and 2 × 10^3^ v/v%, showing varied decay rates, which indicate tunable radiative recombination influenced by ligand-induced changes, aligning with the fast recombination rates and high absorption coefficients (10^5^ to 10^6^ cm^−1^) typical of direct bandgap PQDs. Panel (c) displays PL spectra of PQDs at temperatures of 100 °C, 120 °C, 140 °C, 160 °C, and 180 °C, with emission peaks ranging from 493 to 530 nm, illustrating how temperature affects the bandgap and emission intensity due to quantum confinement and defect tolerance, where defect states within the bands minimize non-radiative recombination for stable fluorescence.^21^ The narrow FWHM (∼20 nm for CsPbBr_3_ at ∼520 nm) ensures high color purity, critical for sensing applications. Panel (d) shows absorbance spectra under the same temperature conditions, revealing shifts in the absorption edge (*e.g.*, ∼3.0 eV for CsPbCl_3_ to ∼1.7 eV for CsPbI_3_) due to size or halide composition tuning, highlighting the large absorption coefficients and efficient light harvesting, which support responsive fluorescence signals for analyte detection (Fig. 2).

#### 2.1.3. Surface chemistry and ligand effects

The surface chemistry of PQDs significantly influences their optical and sensing properties. PQDs are typically capped with organic ligands, such as oleylamine (OAm) or oleic acid (OA), which passivate surface defects, enhance colloidal stability, and prevent aggregation. The ligand type and density affect the PLQY and environmental stability. For instance, long-chain ligands like OAm provide steric stabilization but may hinder analyte access in sensing applications, reducing sensitivity. Conversely, shorter or zwitterionic ligands can improve ion accessibility while maintaining stability in polar media.^18,35^ Surface defects, if unpassivated, can act as non-radiative recombination centers, lowering PLQY and affecting sensing selectivity.^33^ Ligand exchange strategies, such as replacing OAm with zwitterionic molecules, have been explored to enhance water compatibility for aqueous sensing.

#### 2.1.4. Exciton dynamics and sensing relevance

The exciton dynamics in PQDs, characterized by short radiative lifetimes (1–10 ns) and high exciton binding energies (20–100 meV), contribute to their sensitivity in sensing applications. The strong excitonic effects enable efficient energy transfer mechanisms, such as FRET or electron transfer, when PQDs interact with metal ions. For example, the presence of heavy metal ions like Cu^2+^ or Hg^2+^ can induce fluorescence quenching through electron transfer or ion exchange, providing a measurable signal for detection.^14,36^ The high surface-to-volume ratio of PQDs amplifies these interactions, making them highly responsive to low analyte concentrations, often achieving LOD in the nanomolar range.

### 2.2. Types of perovskite quantum dots

PQDs can be broadly classified into lead-based and lead-free variants, each with distinct structural, optical, and practical characteristics. Lead-based PQDs, such as CsPbX_3_ and CH_3_NH_3_PbX_3_, are the most studied due to their superior optical properties, while lead-free PQDs, such as Cs_3_Bi_2_X_9_ and CsSnX_3_, are gaining attention for their reduced toxicity and environmental compatibility.^31,37^

#### 2.2.1. Lead-based perovskite quantum dots

Lead-based PQDs, including inorganic CsPbX_3_ (X = Cl, Br, I) and organic–inorganic hybrids like CH_3_NH_3_PbX_3_ or FAPbX_3_, are renowned for their high PLQY (up to 90%), narrow emission spectra, and tunable bandgaps (1.7–3.0 eV). The lead cation (Pb^2+^) in the B-site contributes to strong spin–orbit coupling, resulting in a direct bandgap and efficient radiative recombination. CsPbX_3_ PQDs are fully inorganic, offering enhanced thermal and chemical stability compared to hybrids. For example, CsPbBr_3_ exhibits robust green emission (∼520 nm) and is widely used for detecting ions like Cu^2+^ and Cd^2+^ due to its high sensitivity to quenching mechanisms.^38^ Organic–inorganic hybrids, such as CH_3_NH_3_PbBr_3_, incorporate organic cations like methylammonium, which increase lattice flexibility but reduce stability under moisture or heat due to the volatility of the organic component. The halide composition in lead-based PQDs enables precise optical tuning, with CsPbCl_3_ emitting blue (410 nm), CsPbBr_3_ green, and CsPbI_3_ red (680 nm). Mixed-halide systems like CsPb(Br/Cl)_3_ allow continuous spectral adjustment, vital for ratiometric sensing.^37,39^ However, Pb^2+^ toxicity poses challenges, necessitating stringent handling and disposal protocols for environmental and biological sensing applications.

#### 2.2.2. Lead-free perovskite quantum dots

Lead-free PQDs, such as Cs_3_Bi_2_X_9_, CsSnX_3_, or MASnX_3_, are developed to address the toxicity concerns of lead-based counterparts. These materials replace Pb^2+^ with less toxic metals like Bi^3+^ or Sn^2+^. Cs_3_Bi_2_X_9_ PQDs adopt a layered or dimer structure rather than the standard ABX_3_ perovskite lattice, resulting in lower PLQY (typically 20–50%) and broader emission spectra (FWHM ∼40–60 nm).^40^ Despite these limitations, they offer improved environmental stability and are suitable for detecting ions like Cu^2+^ or Cr^6+^ in aqueous media. CsSnX_3_ PQDs, while promising, suffer from rapid oxidation of Sn^2+^ to Sn^4+^, leading to reduced stability and PL efficiency.^37^ Doping strategies, such as incorporating Eu^3+^ or Mn^2+^, enhance the optical properties and stability of lead-free PQDs, making them viable for eco-friendly sensing applications.

#### 2.2.3. Doped and hybrid perovskite quantum dots

**Fig. 3:**
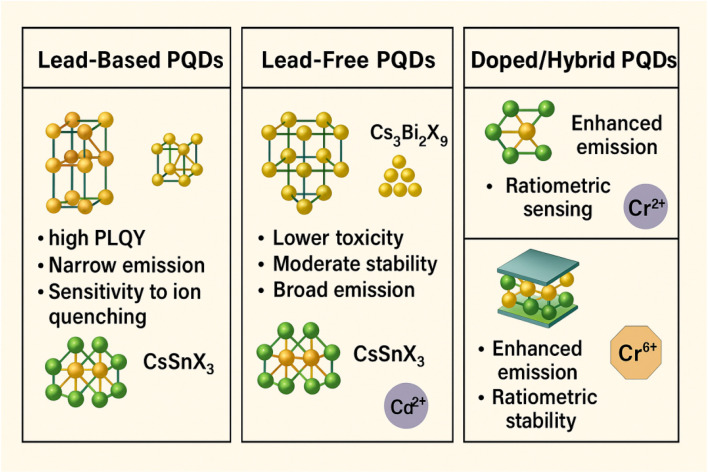
Classification of PQDs into lead-based, lead-free, and doped/hybrid types, highlighting their structural features, optical properties, and sensing capabilities for heavy metal ion detection.

Doping PQDs with transition metal ions (*e.g.*, Mn^2+^, Eu^3+^) or forming hybrid structures (*e.g.*, CsPbBr_3_–MXene) introduces additional functionalities. Mn^2+^-doped CsPbCl_3_ PQDs exhibit dual emission (excitonic and dopant-induced), enabling ratiometric sensing for improved accuracy. Hybrid structures, such as CsPbBr_3_ encapsulated in metal–organic frameworks (MOFs) or combined with MXene, enhance stability and sensitivity by providing a protective matrix or facilitating charge transfer.^41^ These advanced PQDs are particularly effective for detecting multiple analytes, including heavy metal ions, in complex matrices (Fig. 3).

#### 2.2.4. Comparison of PQD types

The diverse classes of PQDs exhibit distinct optical, structural, and stability characteristics, making them suitable for various sensing applications. Lead-based PQDs offer superior PLQY and tunable emission but are hindered by toxicity and environmental concerns. In contrast, lead-free PQDs prioritize eco-friendliness and stability, albeit with compromised optical performance. Doped and hybrid PQDs combine enhanced stability and multifunctionality, enabling advanced sensing capabilities in complex environments. The below table summarizes their key properties to facilitate a comparative understanding (Table 1).

**Table 1:** Comparative overview of perovskite quantum dot types and their properties

PQD type	Composition example	Lead-based/lead-free	PLQY (%)	Emission range (nm)	Stability	Sensing applications	Ref.
Inorganic lead-based	CsPbX_3_ (X = Cl, Br, I)	Lead-based	50–90	410–680	Moderate (sensitive to moisture/UV)	Cu^2+^, Hg^2+^, Cd^2+^ detection	37 and 39
Organic–inorganic hybrid	CH_3_NH_3_PbX_3_, FAPbX_3_	Lead-based	40–80	450–700	Low (volatile organic cations)	Cu^2+^, Fe^3+^ detection	42
Lead-free bismuth-based	Cs_3_Bi_2_X_9_	Lead-free	20–50	400–600	High (water-stable)	Cu^2+^, Cr^6+^ detection	43
Lead-free tin-based	CsSnX_3_, MASnX_3_	Lead-free	10–40	600–800	Low (Sn^2+^ oxidation)	Pb^2+^ detection	44 and 45
Doped/hybrid	CsPbCl_3_:Mn, CsPbBr_3_@MOF	Lead-based/lead-free	30–80	400–700	High (matrix-protected)	Ratiometric sensing, multi-ion detection	41

### 2.3. Synthesis methods and their impact on sensing performance

The synthesis strategy employed for PQDs plays a critical role in determining their particle size, crystallinity, surface chemistry, and ultimately their performance in sensing applications. Controlled synthesis influences the PLQY, surface defect density, ion accessibility, and stability in aqueous media—key parameters governing their suitability for detecting heavy metal ions. This section systematically discusses five major synthesis methods, highlighting their operational principles, advantages, and limitations, as well as their implications for sensor performance.

#### 2.3.1. Hot-injection method

The hot-injection method is one of the most widely used and precise techniques for synthesizing high-quality PQDs. Typically, cesium oleate is rapidly injected into a hot solution (140–200 °C) containing a lead halide precursor (*e.g.*, PbBr_2_) dissolved in a non-coordinating solvent (*e.g.*, octadecene) along with ligands such as oleic acid (OA) and oleylamine (OAm), under an inert atmosphere (*e.g.*, N_2_ or Ar). The sudden introduction of the cesium source induces burst nucleation followed by controlled growth, yielding monodisperse nanocrystals with tunable sizes (2–8 nm) and high crystallinity. This technique allows precise control over the halide composition (Cl^−^, Br^−^, I^−^) and particle size, enabling direct tuning of bandgap and emission wavelength. PQDs synthesized by hot-injection typically exhibit PLQYs above 80% and low surface defect densities.^46,47^ These features contribute to enhanced sensing performance through efficient fluorescence quenching mechanisms such as cation exchange or electron transfer, with LODs reaching sub-nanomolar levels for ions like Cu^2+^ and Hg^2+^. However, the method requires rigorous control of reaction parameters and inert conditions, limiting scalability and water stability unless surface modification or encapsulation is performed.

#### 2.3.2. Hydrothermal and solvothermal synthesis

Hydrothermal (aqueous-based) and solvothermal (organic solvent-based) methods involve sealing precursors in an autoclave and heating to elevated temperatures (100–200 °C) and pressures for several hours. These methods are particularly well-suited for the synthesis of lead-free PQDs, such as Cs_3_Bi_2_X_9_ or CsSnX_3_, offering improved environmental compatibility. The high-pressure environment promotes better ligand coordination, resulting in nanocrystals with moderate crystallinity and PLQYs in the range of 50–70%. Moreover, these methods yield PQDs with enhanced water stability, essential for applications in aqueous sensing.^48,49^ Nevertheless, they often require longer reaction times and complex equipment setups, which can impede throughput and reproducibility.

#### 2.3.3. Room-temperature precipitation

**Fig. 4:**
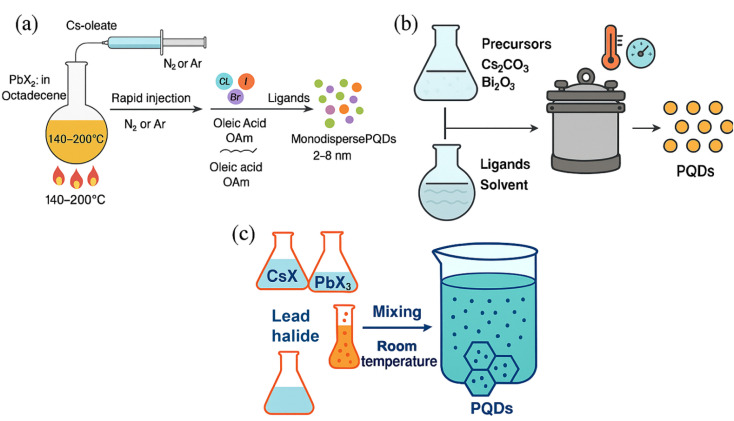
Diverse synthetic routes for perovskite quantum dots: (a) hot-injection method; (b) hydrothermal and solvothermal synthesis procedures; (c) room-temperature precipitation technique.

This method represents the most accessible and energy-efficient approach to PQD synthesis. It typically involves mixing cesium and lead halide salts in low-boiling point solvents such as ethanol or water under ambient conditions. While advantageous for rapid and low-cost production, this approach generally yields PQDs with larger sizes (8–15 nm), poor crystallinity, and high surface defect densities. These features reduce the PLQY (30–50%) and make the resulting nanocrystals less suitable for high-sensitivity detection. Nonetheless, this method finds utility in preliminary screening studies or applications where ultra-trace detection is not required.^50,51^ It is particularly appealing in resource-limited settings where access to sophisticated equipment is restricted (Fig. 4).

#### 2.3.4. Microwave-assisted synthesis

Microwave-assisted synthesis employs dielectric heating to rapidly and uniformly heat reaction mixtures, enabling accelerated nucleation and growth of PQDs. Typically, lead halide and cesium precursors are combined with coordinating ligands and subjected to microwave irradiation for short durations (minutes), resulting in highly crystalline PQDs with size ranges of 3–10 nm and PLQYs between 60–85%. The homogeneous temperature profile minimizes thermal gradients and suppresses defect formation, contributing to enhanced stability and sensitivity. Microwave synthesis is particularly effective for generating PQDs used in ratiometric sensing, such as dual-emission systems for Fe^3+^ and Cr^6+^ detection.^52,53^ However, the method requires specialized microwave reactors and may present challenges in scaling for industrial use.

#### 2.3.5. Ligand-assisted reprecipitation

**Fig. 5:**
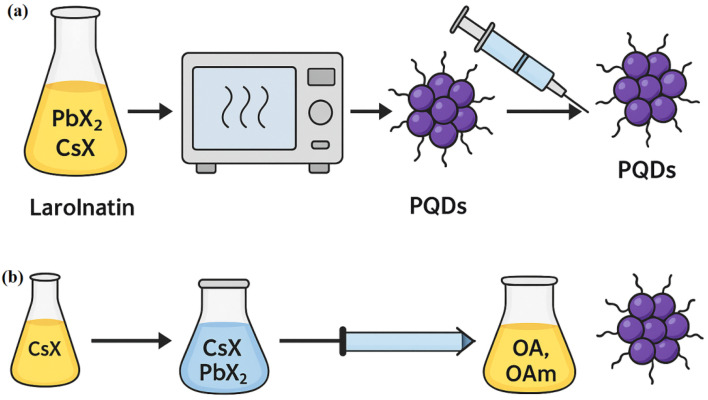
Synthesis approaches for PQDs: (a) microwave-assisted synthesis technique; (b) ligand-assisted reprecipitation method.

LARP is a room-temperature, scalable approach ideal for cost-effective PQD production. In this method, precursors (*e.g.*, CsX and PbX_2_) are dissolved in a polar solvent such as *N*,*N*-dimethylformamide (DMF) and rapidly injected into a non-polar solvent like toluene or hexane containing capping ligands (*e.g.*, OA, OAm). The difference in solubility induces supersaturation and instantaneous nucleation of PQDs. Although LARP-synthesized PQDs typically have broader size distributions (5–12 nm) and moderate PLQY (50–70%), the process is simpler and better suited for large-scale production. The nature and concentration of ligands significantly affect the colloidal stability, ion accessibility, and defect passivation.^54,55^ By incorporating hydrophilic or zwitterionic ligands, water compatibility is improved, enabling application in aqueous sensing environments. However, LARP products often require post-synthetic treatments (*e.g.*, ligand exchange, encapsulation) to enhance long-term stability and minimize surface defects (Fig. 5).

Table 2 provides a comparative overview of commonly used PQD synthesis techniques, highlighting key physicochemical properties and their implications for heavy metal ion detection sensitivity and stability.

**Table 2:** Comparison of synthesis methods for PQDs and their impact on heavy metal ion sensing performance

Synthesis method	Precursor type	Size (nm)	PLQY (%)	Defect density	Ion accessibility	Water compatibility	Scalability	Sensing performance	Ref.
Hot-injection	Cs-oleate + PbX_2_ in OA/OAm	2–8	>80	Very low	Moderate–low	Low (requires treatment)	Low	High sensitivity (<1 nM); efficient quenching	46 and 47
Hydro/solvothermal	BiX_3_ or SnX_2_ + CsX in sealed autoclave	5–12	50–70	Moderate	Moderate	High	Medium	Moderate sensitivity (∼tens of nM); lead-free options	48 and 49
Room-temp precipitation	PbX_2_ + CsX in ethanol/water	8–15	30–50	High	High	High	Very high	Low sensitivity (>μM); fast and inexpensive	50 and 51
Microwave-assisted	Same as hot-injection	3–10	60–85	Low	Moderate	Moderate	Medium	High sensitivity (<5 nM); rapid synthesis	52 and 53
LARP	CsX + PbX_2_ in DMF/toluene	5–12	50–70	Moderate	High (short ligands)	Moderate (hydrophilic ligands)	High	Moderate sensitivity; water-compatible	54 and 55

## 3. Mechanisms of heavy metal ion detection

The detection of heavy metal ions using PQDs leverages their unique optoelectronic properties, including high PLQY, narrow emission spectra, and tunable bandgaps, to achieve sensitive and selective responses to analyte interactions. These responses primarily manifest as changes in PL intensity, such as quenching or, less commonly, enhancement, driven by specific physical and chemical interactions between PQDs and metal ions.^56–58^ This section explores the fundamental mechanisms underlying these interactions, including cation exchange, electron transfer, FRET, surface trap-mediated quenching, and charge transfer complex formation. The critical role of surface ligands in modulating selectivity and sensitivity is also discussed, alongside theoretical insights from computational methods, such as density functional theory (DFT) and molecular dynamics (MD) simulations, to elucidate the electronic and structural factors governing these mechanisms. The focus is on the fundamental processes, avoiding overlap with sensor performance or applications, which are reserved for subsequent sections.

### 3.1. Fluorescence quenching and enhancement mechanisms

The interaction of heavy metal ions with PQDs typically results in fluorescence quenching, where PL intensity decreases due to the creation of non-radiative recombination pathways, or, in rare cases, enhancement, where PL intensity increases due to passivation of surface defects. These changes stem from alterations in the electronic structure, surface chemistry, or energy transfer dynamics of PQDs, driven by distinct mechanisms.^11,59^

#### 3.1.1. Cation exchange

Cation exchange is a dominant mechanism in lead-based PQDs, such as CsPbX_3_ (X = Cl, Br, I) or CH_3_NH_3_PbX_3_, where heavy metal ions replace the B-site cation (typically Pb^2+^) at the PQD surface. This substitution disrupts the perovskite lattice, introducing defects or mid-gap states that facilitate non-radiative recombination, leading to fluorescence quenching. For example, Hg^2+^ ions, with an ionic radius (110 pm) close to Pb^2+^ (119 pm), readily replace Pb^2+^ in CH_3_NH_3_PbBr_3_, causing lattice strain and quenching PL within seconds due to rapid ion exchange kinetics.^59^ The selectivity of this mechanism depends on the ionic radius, charge, and coordination chemistry of the analyte, making it highly effective for Hg^2+^ detection over other ions like Zn^2+^ or Na^+^. In lead-free PQDs, such as Cs_3_Bi_2_X_9_, cation exchange is less prevalent due to the higher stability of Bi^3+^, limiting this mechanism's applicability.^60^ The efficiency of cation exchange is also influenced by surface ligand density, which can either facilitate or hinder ion access to the lattice.^61^

**Fig. 6:**
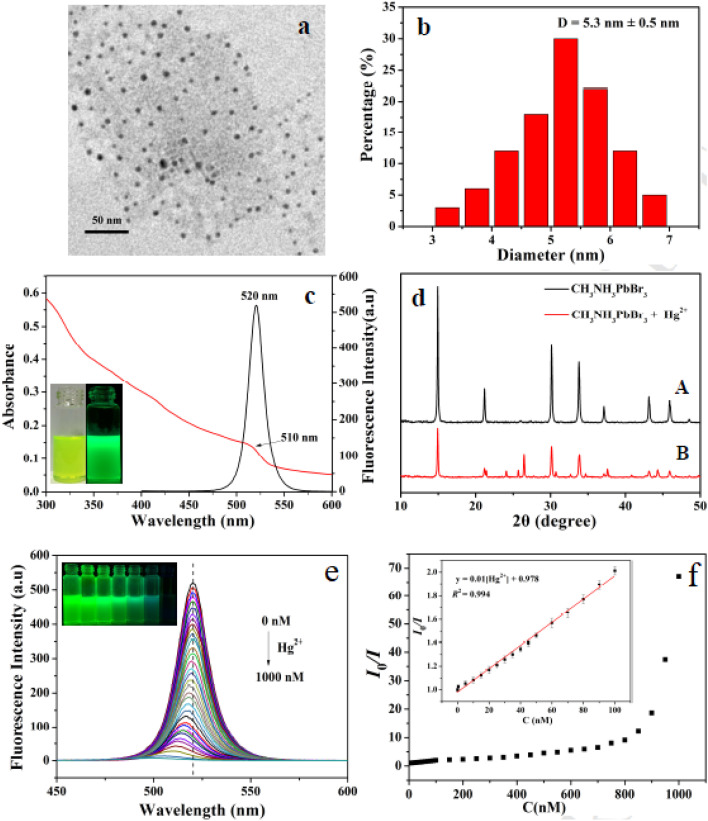
(a) TEM image of CH_3_NH_3_PbBr_3_ QDs, (b) size distribution of CH_3_NH_3_PbBr_3_ QDs, (c) UV-vis and PL spectra of CH_3_NH_3_PbBr_3_ QDs, (d) XRD patterns with/without Hg^2+^, (e) fluorescence changes with varying Hg^2+^, (f) *I*_0_/*I vs.* Hg^2+^ (0–100 nM) linear fit. Reprinted with permission from ref. 59. Copyright 2017, Elsevier.

Panel (a) in Fig. 6 presents a TEM image of CH_3_NH_3_PbBr_3_ QDs, revealing their nanoscale dimensions and uniform distribution, with a scale bar of 50 nm, consistent with the 2–10 nm range where quantum confinement effects dominate.^59^ Panel (b) shows the size distribution analysis, indicating an average diameter of 5.3 ± 0.5 nm, which influences the discrete energy levels and size-dependent bandgap, enhancing photoluminescence properties critical for sensing applications. These structural characteristics support the cation exchange mechanism, where the surface accessibility of Pb^2+^ (ionic radius 119 pm) facilitates rapid ion substitution by analytes like Hg^2+^ (110 pm), disrupting the lattice and introducing defects. Panel (c) displays UV-vis absorption and photoluminescence emission spectra of CH_3_NH_3_PbBr_3_ QDs, with an absorption edge around 520 nm and a sharp emission peak at 510 nm, reflecting the high oscillator strength and narrow FWHM (12–40 nm) due to quantum confinement. The inset photograph confirms the green emission, which is quenched upon Hg^2+^ exchange due to non-radiative recombination induced by lattice strain. Panel (d) presents XRD patterns of CH_3_NH_3_PbBr_3_ QDs in the absence and presence of Hg^2+^, showing peak shifts that indicate lattice disruption and mid-gap states formation, highlighting the selectivity and rapid kinetics of cation exchange for Hg^2+^ detection over other ions. Panel (e) illustrates the evolution of fluorescence spectra of CH_3_NH_3_PbBr_3_ QDs with increasing Hg^2+^ concentrations (0 to 1000 nM), showing a progressive quenching of the 510 nm emission peak, consistent with cation exchange disrupting the perovskite lattice and enhancing non-radiative recombination. Panel (f) provides a linear fitting curve of *I*_0_/*I versus* Hg^2+^ concentration (0–100 nM), with a correlation coefficient (*R*^2^ = 0.994), demonstrating the sensitivity and selectivity of this mechanism for Hg^2+^ detection, influenced by ionic radius compatibility and surface ligand density that modulates ion access.

#### 3.1.2. Electron transfer

Electron transfer occurs when heavy metal ions interact with the PQD surface, enabling the transfer of electrons from the conduction band of the PQD to the ion's unoccupied orbitals or *vice versa*, creating non-radiative recombination pathways that quench fluorescence. For instance, Cu^2+^ ions, with their partially filled d-orbitals, act as electron acceptors in CsPbBr_3_ PQDs, reducing PL intensity by accepting electrons from the conduction band.^62^ This mechanism is particularly effective for transition metal ions like Cu^2+^, Fe^3+^, or Cr^6+^, whose redox potentials align with the PQD's band structure, facilitating efficient electron transfer.^62,63^ The quenching efficiency depends on the energy level alignment between the PQD's conduction band and the ion's orbitals, as well as the accessibility of the PQD surface, which is modulated by ligands. Electron transfer is more pronounced in organic solvents, where ligands like OAm allow ion access, but may be limited in aqueous media due to ligand barriers.^61^

#### 3.1.3. Förster resonance energy transfer

FRET involves non-radiative energy transfer from the excited state of a PQD (donor) to a heavy metal ion or a nearby acceptor molecule, resulting in fluorescence quenching. FRET requires spectral overlap between the PQD's emission and the acceptor's absorption spectrum, as well as proximity within 10 nm. For example, in a ratiometric sensor, CsPbBr_3_ PQDs transfer energy to gold nanoclusters in the presence of Cu^2+^, quenching the PQD's green emission (∼520 nm) while enhancing the nanocluster's emission due to spectral overlap.^64^ FRET is highly sensitive but less selective, as multiple analytes may induce similar energy transfer effects, necessitating careful sensor design to ensure specificity.^65^ The efficiency of FRET depends on the dipole–dipole coupling strength, which is influenced by the PQD's emission properties and the ligand-mediated distance between the PQD and the acceptor.^66^

#### 3.1.4. Surface trap-mediated quenching

Surface trap-mediated quenching occurs when heavy metal ions interact with surface defects or trap states on PQDs, such as uncoordinated halide ions or underpassivated cation sites, creating non-radiative recombination centers that quench fluorescence. For instance, Fe^3+^ ions bind to surface defects on CsPbBr_3_–CsPb_2_Br_5_ PQDs, forming trap states that capture excitons and reduce PL intensity.^67^ This mechanism is particularly relevant for PQDs with high surface-to-volume ratios, where defects are more prevalent. In lead-free PQDs like MASnBr_3_, surface trap-mediated quenching is enhanced by the interaction of ions like Cr^6+^ with undercoordinated Sn^2+^ sites, leading to efficient quenching.^68^ The extent of quenching depends on the defect density and ligand passivation, which can either suppress or amplify trap-mediated effects. Surface modification with ligands like APTES can reduce defect density, mitigating non-specific quenching and enhancing selectivity.^67^

#### 3.1.5. Charge transfer complex formation

Charge transfer complex formation involves the creation of a transient complex between the PQD surface and a heavy metal ion, leading to fluorescence quenching through the formation of a charge transfer state. This mechanism is driven by the coordination of metal ions with surface ligands or lattice ions, forming a complex that alters the electronic structure of the PQD. For example, Cu^2+^ ions can form coordination complexes with amine groups on CsPbBr_3_ PQDs, creating a charge transfer state that quenches PL by redirecting excitons to non-radiative pathways.^69^ This mechanism is particularly effective for ions with strong coordination abilities, such as Cu^2+^ or Cd^2+^, and is enhanced in heterostructures like CsPbBr_3_–Ti_3_C_2_T_*x*_ MXene, where the MXene facilitates charge transfer.^70^ The stability and quenching efficiency of the complex depend on the ligand's coordination strength and the ion's electronegativity.

**Fig. 7:**
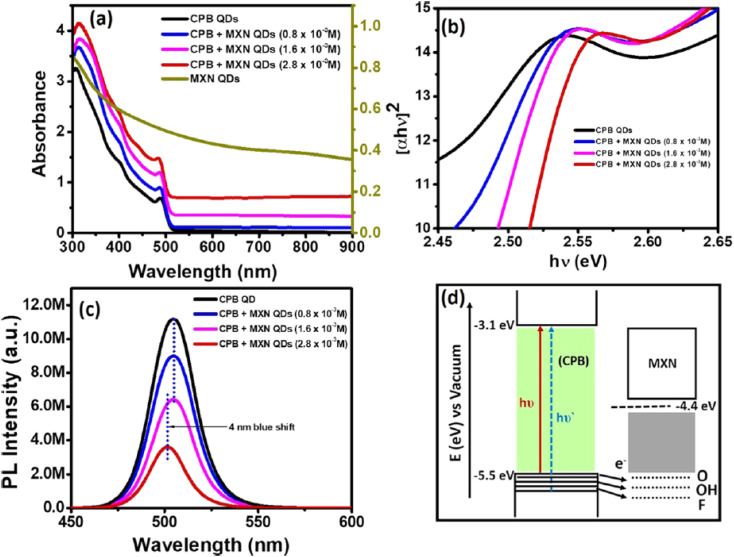
(a) Absorbance spectra of CsPbBr_3_ QDs, MXN QDs, and CPB-MXN composites, (b) Tau plots of CPB-MXN *vs.* pure CsPbBr_3_, (c) PL spectra of CPB-MXN with MXN concentrations 0.8 × 10^−3^, 1.6 × 10^−3^, 2.8 × 10^−3^ M, (d) schematic of CPB-MXN band alignment. CPB QD concentration is constant (1.9 × 10^−7^ M). Reprinted with permission from ref. 70. Copyright 2020, American Chemical Society.

Panel (a) in Fig. 7 presents absorbance spectra of CsPbBr_3_ QDs (black line), MXN QDs (dark yellow), and CsPbBr_3_ QD–Ti_3_C_2_T_*x*_ QD (CPB-MXN) composites, showing distinct absorption edges influenced by the formation of charge transfer complexes, where heavy metal ions coordinate with surface ligands, altering the electronic structure [ref. 70]. Panel (b) in Fig. 7 displays tau plots of the CPB-MXN composite compared to pure CsPbBr_3_, indicating faster decay rates due to charge transfer states that redirect excitons to non-radiative pathways. Panel (c) shows steady-state PL spectra at 410 nm excitation for the CPB-MXN composite with varying MXN concentrations (0.8 × 10^−3^, 1.6 × 10^−3^, 2.8 × 10^−3^ M), revealing a 4 nm blue shift and quenching, consistent with the transient complex formation that enhances non-radiative recombination, particularly with ions like Cu^2+^ coordinating with amine groups. Panel (d) provides a schematic diagram of the CPB-MXN band alignment, illustrating the energy levels where the charge transfer state forms, with a vacuum level at −3.1 eV and a bandgap of 2.4 eV for CPB, facilitating efficient charge transfer in heterostructures like CsPbBr_3_–Ti_3_C_2_T_*x*_ MXene. This alignment supports the quenching mechanism driven by strong coordination abilities of ions such as Cu^2+^ or Cd^2+^, enhanced by the MXene's role in facilitating charge transfer. The stability and efficiency of this quenching depend on the ligand's coordination strength and the ion's electronegativity, aligning with the mechanism's effectiveness in altering the PQD's electronic structure for sensing applications.

### 3.2. Role of surface ligands in selectivity and sensitivity

Surface ligands, such as OAm, OA, PEI, hydroxypropyl chitosan, or APTES, are critical in modulating the selectivity and sensitivity of PQD-based detection. Ligands passivate surface defects, stabilize colloidal dispersions, and mediate ion–PQD interactions, influencing the efficiency of quenching or enhancement mechanisms. Long-chain ligands like OAm and OA provide steric hindrance, reducing non-specific interactions and enhancing selectivity for ions like Hg^2+^ or Cu^2+^, which penetrate the ligand shell through coordination or ion exchange.^61^ For example, OAm-capped CsPbBr_3_ PQDs exhibit high selectivity for Cu^2+^ due to the ion's ability to coordinate with amine groups, facilitating electron transfer or complex formation.^62^ However, dense ligand layers may reduce sensitivity by limiting ion access, particularly in aqueous media. Hydrophilic or zwitterionic ligands, such as hydroxypropyl chitosan, improve water compatibility and ion accessibility, enhancing sensitivity for ions like Cr^6+^.^71^ PEI-modified MASnBr_3_ PQDs introduce chelating groups that selectively bind Fe^3+^, forming stable complexes that enhance quenching specificity.^68^

Dynamic ligand exchange, where metal ions displace weakly bound ligands, exposes the PQD surface, amplifying quenching through cation exchange or trap-mediated mechanisms. For instance, Cd^2+^ detection with CsPbBr_3_–CsPb_2_Br_5_ PQDs is enhanced by ammonia hydroxide, which reduces ligand density and increases surface accessibility.^72^ Conversely, strongly bound ligands like APTES improve stability and selectivity for Fe^3+^ by minimizing non-specific interactions.^67^

### 3.3. Theoretical insights

Theoretical studies, particularly DFT calculations using functionals like PBE or HSE06 for accurate band structures, provide quantitative insights into the electronic, structural, and kinetic changes induced by heavy metal ions in PQDs. For cation exchange, DFT simulations of Pb^2+^ substitution with Hg^2+^ in CH_3_NH_3_PbBr_3_ reveal the formation of mid-gap states positioned 0.5–1.0 eV below the conduction band (CB, typically at −3.5 eV *vs.* vacuum), which promote non-radiative recombination by increasing the rate constant from ∼10^7^ s^−1^ (pristine) to ∼10^9^ s^−1^, as evidenced by shortened PL lifetimes from 10 ns to ∼2 ns.^59^ The thermodynamic rationale is rooted in a favorable Gibbs free energy change (Δ*G* ≈ −20 to −25 kJ mol^−1^), calculated from the low lattice distortion energy due to similar ionic radii (Pb^2+^: 119 pm; Hg^2+^: 110 pm) and a reduced activation barrier (∼0.2 eV), enabling rapid exchange kinetics on the order of seconds in organic solvents. This mechanism is particularly selective for Hg^2+^, with Δ*G* more negative than for mismatched ions like Zn^2+^ (Δ*G* > −10 kJ mol^−1^), aligning with experimental quenching efficiencies >90%.^59^

Similarly, for electron transfer, Cu^2+^ binding to CsPbBr_3_ surfaces reduces the bandgap by shifting the CB edge downward by 0.3–0.4 eV (from −3.8 eV to −4.1 eV), facilitating efficient electron donation from the PQD CB to Cu^2+^ unoccupied d-orbitals (LUMO at ∼−4.0 eV), with transfer rates ∼5 × 10^10^ s^−1^—significantly faster than other mechanisms like FRET (∼10^8^ s^−1^).^62^ Thermodynamically, this is driven by a Δ*G* ≈ −15 to −18 kJ mol^−1^, arising from redox potential alignment (Cu^2+^/Cu^+^*E*° = 0.15 V *vs.* NHE, matching PQD CB at ∼−0.5 V), which lowers the energy barrier for charge separation and enhances quenching in transition metal detections. In lead-free Cs_3_Bi_2_Br_9_, DFT indicates Cu^2+^ interactions with Bi^3+^ sites create shallow trap states at 0.2–0.3 eV above the valence band (VB at −5.5 eV), increasing non-radiative rates to ∼8 × 10^8^ s^−1^ with a Δ*G* ≈ −12 to −14 kJ mol^−1^, though kinetics are slower due to higher lattice rigidity (activation energy ∼0.4 eV) compared to lead-based systems.^60^ This highlights a trade-off: lead-free PQDs offer eco-friendly thermodynamics but reduced kinetic efficiency for aqueous sensing.

Bandgap effects are central to all detection mechanisms, where metal ions shift band edges and alter recombination pathways. For surface trap-mediated quenching, Fe^3+^ ions induce a redshift in the bandgap of CsPbBr_3_–CsPb_2_Br_5_ by 0.15–0.2 eV (from 2.4 eV to 2.2 eV), driven by strong electrostatic binding (energy ∼ −1.5 to −2.0 eV), which creates defect states and reduces radiative lifetimes from 10–15 ns to 1–3 ns, corresponding to rate increases to 6 × 10^8^ s^−1^.^67^ Thermodynamically, Δ*G* ≈ −19 kJ mol^−1^ favors trap formation due to Fe^3+^ coordination with underpassivated Br^−^ sites, making this mechanism kinetically slower than electron transfer but highly selective in aqueous matrices with pH-dependent interferences. DFT also elucidates ligand roles, showing that ligand–metal coordination (*e.g.*, OAm or PEI with Cu^2+^) lowers electron transfer barriers by 0.3–0.5 eV, increasing rates by 10–100-fold and Δ*G* by −5 to −10 kJ mol^−1^ through stabilized charge-separated states.^73^ This is particularly relevant for ratiometric designs, where FRET efficiencies reach 60–70% with dipole–dipole distances < 10 nm, but kinetics lag (∼10^8^ s^−1^) due to spectral overlap requirements (>50% emission–absorption match) and less negative Δ*G* (∼−10 kJ mol^−1^) compared to direct transfer.

MD simulations further model ion kinetics, revealing that diffusion through the ligand shell (*e.g.*, oleylamine chains ∼ 1–2 nm thick) is rate-limiting, with diffusion coefficients ∼5 × 10^−10^ to 10^−9^ m^2^ s^−1^ and residence times ∼10–100 ps, affecting overall response times (seconds in experiments).^74^ Comparatively, electron transfer exhibits the fastest kinetics (10^10^ s^−1^, low barriers) and most negative Δ*G* for rapid, sensitive detection of transition metals like Cu^2+^; cation exchange offers intermediate rates (10^9^ s^−1^) with high thermodynamic stability for Hg^2+^-specific sensing; while trap-mediated and FRET mechanisms provide tunable selectivity but slower dynamics (10^8^ s^−1^) due to defect/spectral dependencies. These quantitative metrics, validated by hybrid DFT-MD approaches, guide rational PQD design—*e.g.*, ligand engineering to optimize barriers or doping to align band edges—for enhanced sensitivity (sub-nM LODs) and selectivity in diverse matrices.

## 4. PQD-based nanosensors for heavy metal ion detection

PQDs have emerged as highly effective platforms for detecting heavy metal ions, leveraging their exceptional optoelectronic properties, including high PLQY, tunable emission wavelengths, and large surface-to-volume ratios. These attributes enable the development of sensors with superior sensitivity and selectivity, particularly for industrial applications where precise detection of heavy metal contaminants is critical.^11,38^ This section reviews the design of PQD-based nanosensors for specific heavy metal ions, evaluates their performance metrics, compares lead-based and lead-free PQDs, explores strategies for enhancing sensor performance, and details their applications in industrial settings for heavy metal detection. The focus is on practical sensor design and performance, distinct from the mechanistic insights covered previously, ensuring a comprehensive and application-oriented analysis tailored to the detection of heavy metal ions.

### 4.1. Design of PQD-based nanosensors for specific heavy metal ions

The design of PQD-based nanosensors is tailored to detect specific heavy metal ions, including Hg^2+^, Cu^2+^, Cd^2+^, Fe^3+^, Cr^6+^, and Pb^2+^, by exploiting ion-specific interactions with the PQD surface or lattice. For Hg^2+^ detection, CH_3_NH_3_PbBr_3_ PQDs have been developed with a limit of detection (LOD) of 0.124 nM, capitalizing on their high PLQY (50.28%) and efficient surface interactions that lead to fluorescence quenching.^59^ The sensor design leverages the chemical affinity of Hg^2+^ for the perovskite lattice, enabling rapid and selective detection in organic media. Cu^2+^ detection is a focal point, with CsPbBr_3_ PQDs achieving an LOD of 0.1 nM in organic solvents like hexane, benefiting from fast response times (<10 s) and high selectivity over competing ions such as Na^+^ or Zn^2+^.^62^ The design incorporates ligands like OAm to facilitate ion access in non-polar environments. Cd^2+^ detection is achieved using CsPbBr_3_–CsPb_2_Br_5_ PQDs modified with ammonia hydroxide, which reduces ligand density to enhance surface accessibility, resulting in an LOD of 10^−6^ M.^72^ This modification improves sensitivity in aqueous media, critical for industrial wastewater analysis. Lead-free PQDs, such as MASnBr_3_, are designed for Fe^3+^ and Cr^6+^ detection, achieving nanomolar sensitivity through tailored ligand systems like PEI, which forms selective complexes with these ions.^68^ Cs_3_Bi_2_Br_9_ PQDs are engineered for Cu^2+^ detection in aqueous environments, achieving an LOD of 98.3 nM due to their high photostability and ligand-assisted interactions.^60^ For Pb^2+^ detection, CsSnX_3_ PQDs are optimized for non-aqueous media like lubricants, achieving an LOD of 3.5 nM through synthesis methods that enhance PL stability.^75^ These designs highlight the adaptability of PQDs to diverse ions and matrices, driven by tailored material compositions and surface chemistries.

**Fig. 8:**
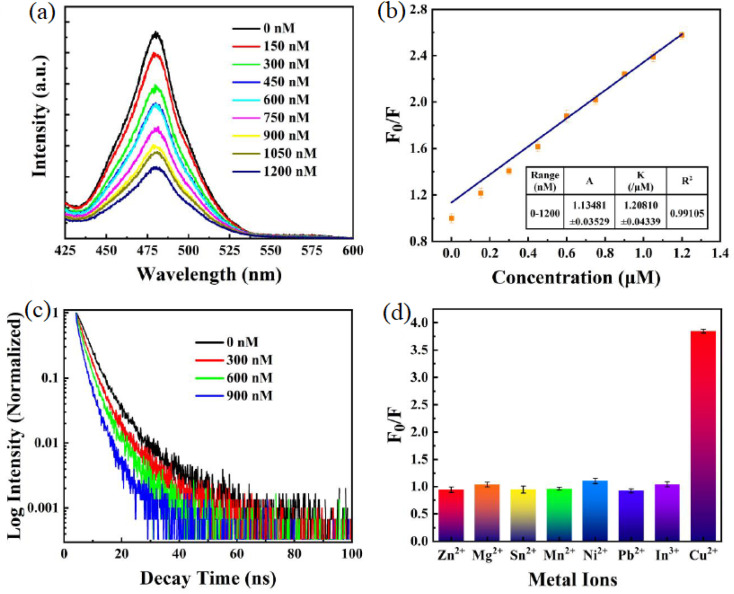
(a) PL intensity of Cs_3_Bi_2_Br_9_ PQDs at different Cu^2+^ concentrations, (b) calibration curve of PL intensity *versus* Cu^2+^ concentration, (c) time-resolved fluorescence profiles with varying Cu^2+^ levels, (d) PL responses of the sensor to various metal ions. Adapted with permission from ref. 60. Copyright 2023, MDPI.

Fig. 8a shows the PL intensity of Cs_3_Bi_2_Br_9_ PQDs at varying Cu^2+^ concentrations (0 to 1200 nM), with a clear quenching trend, reflecting the high PLQY (50.28%) and efficient surface interactions that enable a low LOD of 0.1 nM in organic solvents like hexane, as designed for Cu^2+^ detection. Panel (b) presents a fitting curve for PL intensity *versus* Cu^2+^ concentration, demonstrating a linear range of 0–1200 nM with an *R*^2^ of 0.99105, highlighting the sensor's selectivity and rapid response (<10 s) over competing ions like Na^+^ or Zn^2+^, facilitated by ligands such as OAm. Panel (c) displays time-resolved fluorescence spectra at varying Cu^2+^ concentrations, showing decay time reductions that underscore the fast ion-specific interactions with the PQD lattice, aligning with the tailored design for heavy metal ion sensing. Panel (d) illustrates the PL responses (*F*_0_/*F*) of the probe to different metal ions (Zn^2+^, Mg^2+^, Sn^2+^, Mn^2+^, Ni^2+^, Pb^2+^, In^3+^, Cu^2+^), with Cu^2+^ exhibiting the highest quenching efficiency (*F*_0_/*F* ≈ 3.5), confirming the sensor's selectivity and nanomolar sensitivity (LOD 0.1 nM) for Cu^2+^ in organic media. This design leverages the chemical affinity of Cu^2+^ for the perovskite surface, enhanced by ligand-assisted interactions, consistent with the adaptability of PQDs like Cs_3_Bi_2_Br_9_ for aqueous detection (LOD 98.3 nM) or CH_3_NH_3_PbBr_3_ for Hg^2+^ (LOD 0.124 nM), and CsSnX_3_ for Pb^2+^ (LOD 3.5 nM) in non-aqueous matrices, showcasing tailored surface chemistries for diverse ion-specific applications.

### 4.2. Performance metrics of PQD-based sensors

The performance of PQD-based nanosensors is evaluated through key metrics: sensitivity, selectivity, LOD, and linear detection range, all of which are critical for their practical application in industrial and environmental settings. These metrics are heavily influenced by the matrix in which detection occurs (*e.g.*, organic solvents like hexane, aqueous solutions, or complex media like seawater and wastewater), necessitating standardized calibration protocols, blank measurements, and interference assessments to ensure reproducibility and comparability.

Sensitivity is defined as the change in PL intensity per unit analyte concentration (Δ*I*/Δ*C*, typically in a.u./nM). Lead-based PQDs, such as CsPbX_3_ (X = Cl, Br, I), exhibit high sensitivity due to their strong fluorescence (PL quantum yield, PLQY, 50–90%) and large absorption coefficients (10^5^ to 10^6^ cm^−1^). For instance, CsPbBr_3_ PQDs detect Cu^2+^ in hexane with a sensitivity of ∼10^3^ a.u./nM across a linear range of 0–1200 nM, calibrated using Stern–Volmer plots (*I*_0_/*I vs.* [Cu^2+^]) with blank hexane solutions to correct for background fluorescence.^62^ In aqueous matrices, sensitivity often decreases due to matrix effects; for example, Cs_3_Bi_2_Br_9_ in seawater shows a sensitivity of ∼10^2^ a.u./nM for Cu^2+^, attributed to ionic strength (*e.g.*, 0.6 M NaCl) and pH variations (pH 7.5–8.5), requiring calibration with seawater blanks.^60^

Selectivity is achieved through specific interactions between PQDs and target ions, such as cation exchange for Hg^2+^ or chelation for Fe^3+^, minimizing interference from common ions like K^+^, Na^+^, or Ca^2+^. For example, CH_3_NH_3_PbBr_3_ PQDs selectively detect Hg^2+^ in organic solvents with >95% selectivity over Zn^2+^ and Mg^2+^, confirmed through interference tests where competing ions caused <5% PL change.^59^ In complex matrices like wastewater (pH ∼ 6.5, high Ca^2+^/Mg^2+^), selectivity may drop to ∼80% unless ligand modifications (*e.g.*, polyethylenimine, PEI) enhance ion-specific binding.^68^ Calibration protocols incorporate blank measurements in the respective matrix to establish baseline PL and interference tests to quantify selectivity (*e.g.*, PL quenching <10% for non-target ions).

LOD varies significantly with matrix and calibration methodology. In organic solvents like hexane, where ionic interferences are minimal, LODs reach sub-nanomolar levels (*e.g.*, 0.1 nM for Cu^2+^ with CsPbBr_3_, determined *via* Stern–Volmer with 3*σ*/slope, where *σ* is the standard deviation of blank hexane fluorescence^62^). In contrast, aqueous matrices like seawater or wastewater yield higher LODs (*e.g.*, 98.3 nM for Cu^2+^ with Cs_3_Bi_2_Br_9_ in seawater, calibrated *via* linear *I*_0_/*I* fitting with seawater blanks and interference tests showing ∼15% quenching from Ca^2+^/Mg^2+^ (ref. 60)). Complex matrices introduce challenges like ionic strength, pH fluctuations, and organic matter, which elevate LODs; for instance, wastewater (COD ∼ 200 mg L^−1^) increases LODs to ∼500 nM for Fe^3+^ with MASnBr_3_ due to competing quenching.^68^ Ratiometric sensors, such as CsPbBr_3_ paired with Au nanoclusters, mitigate matrix effects by using dual-emission ratios, achieving LODs of ∼1 nM for Cu^2+^ in aqueous systems with calibration against blank water and <10% interference from Na^+^/K^+^.^64^

Linear detection range typically spans 10^−9^ to 10^−3^ M, depending on the matrix and PQD type. In organic solvents, CsPbBr_3_ detects Cu^2+^ linearly from 0–1200 nM, with high *R*^2^ (>0.99) in Stern–Volmer plots.^62^ In seawater, Cs_3_Bi_2_Br_9_ maintains linearity from 0–1000 nM but requires ligand passivation to counter matrix interferences.^60^ Wastewater matrices narrow the range (*e.g.*, 10–500 nM for Fe^3+^ with MASnBr_3_) due to pH and organic matter effects, calibrated *via* time-resolved decay to isolate analyte-specific quenching.^68^

To standardize comparisons across studies, Table 3 summarizes LODs, matrices, calibration protocols, blank measurements, interference tests, selectivity metrics, and linear ranges. Organic solvents generally yield lower LODs and wider ranges due to reduced interferences, while aqueous and complex matrices require robust calibration (*e.g.*, matrix-matched blanks) and ligand strategies to maintain performance. These insights guide sensor optimization for specific applications, such as trace metal detection in environmental monitoring.

**Table 3:** Summary of LODs and performance metrics for PQD-based sensors

PQD type	Ion	LOD	Matrix	Calibration protocol	Blanks/interferences	Selectivity	Linear range	Ref.
CsPbBr_3_	Cu^2+^	0.1 nM	Hexane	Stern–Volmer (3*σ*/slope)	Hexane blanks; <5% quenching by Na^+^, Zn^2+^	>95%	0–1200 nM	62
Cs_3_Bi_2_Br_9_	Cu^2+^	98.3 nM	Seawater	Linear *I*_0_/*I* fitting	Seawater blanks; ∼15% quenching by Ca^2+^, Mg^2+^ (ligand-mitigated)	∼80%	0–1000 nM	60
CH_3_NH_3_PbBr_3_	Hg^2+^	0.124 nM	Organic solvent	*I*_0_/*I vs.* concentration (3*σ*/slope)	Solvent blanks; <5% quenching by Zn^2+^, Mg^2+^	>95%	0–1000 nM	59
MASnBr_3_	Fe^3+^	∼1 nM	Aqueous	Time-resolved decay (3*σ*/slope)	Water blanks; ∼10% quenching by Cr^6+^ (PEI-selective)	∼90%	10^−9^ to 10^−5^ M	68
CsPbBr_3_@MOF	Cu^2+^	1.63 nM	Hexane	Fluorescence quenching plot	Hexane blanks; <8% quenching by K^+^, Ca^2+^	>92%	10^−9^ to 10^−6^ M	78
CsPbBr_3_–CsPb_2_Br_5_	Cd^2+^	1 μM	Wastewater	Stern–Volmer (3*σ*/slope)	Wastewater blanks; ∼20% quenching by Cu^2+^ (ligand-modified)	∼75%	10–500 nM	72

### 4.3. Comparison of lead-based and lead-free PQDs for heavy metal ion detection

Lead-based PQDs, such as CsPbX_3_ (X = Cl, Br, I) and CH_3_NH_3_PbX_3_, are highly effective for detecting heavy metal ions due to their superior optoelectronic properties, including high photoluminescence quantum yield and narrow emission spectra. These attributes enable ultra-low LODs, often below 10 nM, for ions like Hg^2+^ and Cu^2+^. For instance, CH_3_NH_3_PbBr_3_ PQDs achieve an LOD of 0.124 nM for Hg^2+^, with rapid response times (<10 s) and high sensitivity, making them suitable for environmental monitoring.^59^ Similarly, CsPbBr_3_ PQDs in organic solvents detect Cu^2+^ with an LOD of 0.1 nM, leveraging OAm ligands for enhanced specificity.^62^

**Fig. 9:**
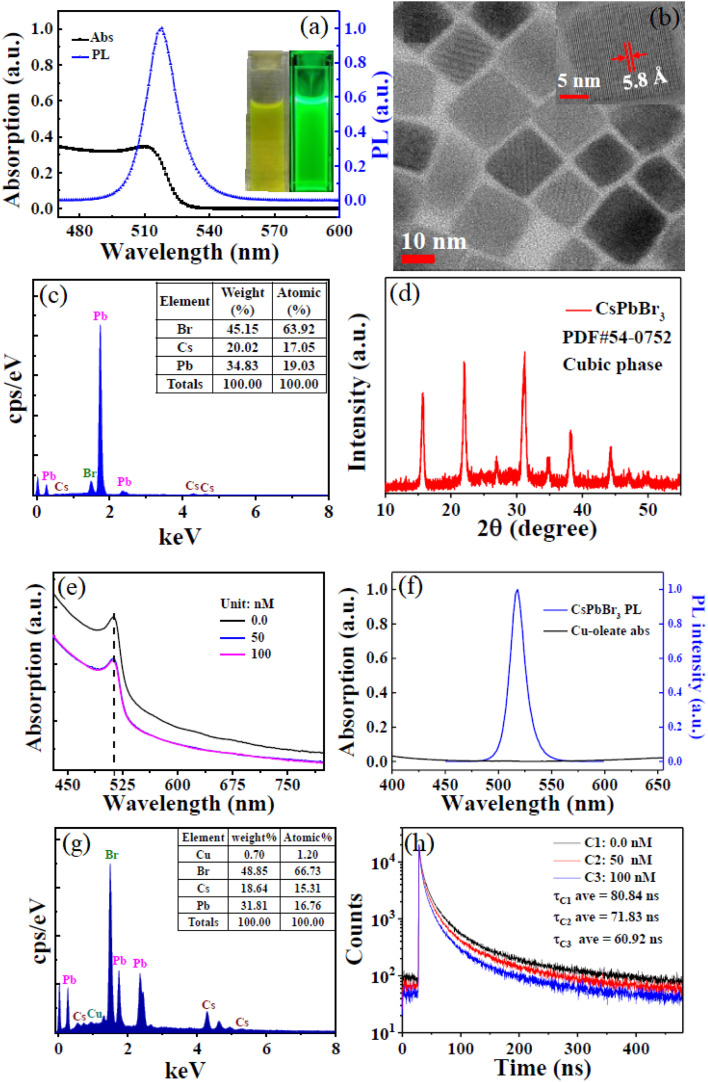
Characterizations of CsPbBr_3_ PQDs: (a) absorption and PL spectra, inset: images under daylight and 365 nm; (b) TEM and HRTEM inset; (c) EDS spectrum, inset: elemental ratios; (d) XRD pattern; (e) absorption spectra with Cu^2+^; (f) PL spectrum with Cu-oleate; (g) EDS of aggregates, inset: ratios; (h) time-resolved PL decay, inset: lifetimes. Adapted with permission from ref. 62. Copyright 2018, Royal Society of Chemistry.

The characterization of CsPbBr_3_ PQDs, as depicted in Fig. 9, provides a comprehensive analysis of their structural and optical properties.^62^ Panels (a) and (f) present the absorption and PL spectra, respectively, showing a strong absorption peak around 510 nm and a corresponding PL emission peak, indicating the high optical quality of the PQDs dispersed in hexane under daylight and 365 nm UV lamp irradiation. The inset images in (a) visually confirm the uniform dispersion, while panel (b) offers a TEM image with a high-resolution TEM (HRTEM) inset, revealing a lattice spacing of 5.8 Å, consistent with the cubic phase of CsPbBr_3_. These structural insights underscore the PQDs' crystallinity and potential for optoelectronic applications. Elemental composition and crystallographic structure are further elucidated in panels (c) and (d). Panel (c) displays the energy-dispersive X-ray spectroscopy (EDS) spectrum with an inset table detailing the atomic percentages (Cs: 20.2%, Pb: 34.83%, Br: 45.15%), confirming the stoichiometric ratio of the PQDs. Panel (d) shows the XRD pattern, matching the PDF # 54-0752 reference for the cubic phase, which aligns with the HRTEM findings and validates the phase purity of the synthesized CsPbBr_3_ PQDs. These results highlight the material's compositional integrity and crystalline order, critical for its performance in heavy metal ion detection.

The comparison of CsPbBr_3_ PQDs with and without Cu^2+^ ions is illustrated in panels (e) and (f). Panel (e) exhibits absorption spectra of CsPbBr_3_ PQDs at different Cu^2+^ concentrations (0, 50, 100 nM), showing a gradual decrease in absorption intensity with increasing Cu^2+^, indicative of ion-induced quenching. Panel (f) compares the PL spectra of pristine CsPbBr_3_ PQDs and those with Cu-oleate, revealing a significant PL intensity drop upon Cu^2+^ addition, with an inset showing elemental ratios post-incubation. This quenching behavior is key to achieving ultra-low LODs, such as 0.1 nM for Cu^2+^, as noted in the context of environmental monitoring applications. Panels (g) and (h) provide additional spectroscopic and kinetic data. Panel (g) presents the EDS spectrum of CsPbBr_3_ aggregates with an inset table (Br: 48.35%, Cs: 18.64%, Pb: 31.81%), confirming the elemental composition after Cu^2+^ interaction. Panel (h) displays time-resolved PL decay curves at different Cu^2+^ concentrations (0, 50, 100 nM), with average lifetimes decreasing from 80.84 ns to 60.92 ns, reflecting enhanced non-radiative recombination due to Cu^2+^. These kinetic changes correlate with the rapid response times (<10 s) and high sensitivity observed in lead-based PQDs for detecting heavy metal ions like Cu^2+^, reinforcing their efficacy in practical sensing applications.

These systems excel in applications requiring high sensitivity, such as industrial solvent analysis. However, lead-based PQDs face significant challenges due to the toxicity of Pb^2+^, which limits their use in environmentally sensitive applications. Their stability in aqueous media is often poor, necessitating advanced stabilization techniques. For example, CsPbBr_3_ PQDs encapsulated in PCN-333(Fe) MOFs achieve an LOD of 1.63 nM for Cu^2+^ with improved stability and a 6.5-fold PL enhancement, suitable for water quality monitoring.^78^ Encapsulation in ZIF-8 MOFs further extends stability to 15 days in aqueous solutions, with an LOD of 2.64 nM for Cu^2+^.^79^ Phase transfer methods using OAm also enable aqueous detection of Cu^2+^, though complex synthesis and lead toxicity remain barriers to scalability.^61^

Lead-free PQDs, such as Cs_3_Bi_2_X_9_ and MASnBr_3_, offer environmentally friendly alternatives with enhanced stability in aqueous environments, critical for applications like wastewater and seawater analysis. Eu^3+^-doped Cs_3_Bi_2_Br_9_ PQDs detect Cu^2+^ with an LOD of 98.3 nM in seawater, benefiting from high photostability and moisture resistance.^60^ Cs_3_Bi_2_Cl_9_ PQDs, passivated with hydroxypropyl chitosan, achieve an LOD of 0.27 μM for Cr^6+^ in wastewater, supported by ligand-enhanced sensitivity.^71^ These lead-free systems are ideal for eco-friendly industrial applications, particularly in marine and environmental monitoring. Despite their environmental advantages, lead-free PQDs typically exhibit lower PLQY (20–50%) and broader emission spectra, leading to higher LODs compared to lead-based systems. For example, MASnBr_3_ PQDs with PEI ligands detect Fe^3+^ and Cr^6+^ in the nanomolar range but face stability challenges in harsh conditions.^68^ CsSnX_3_ PQDs achieve a low LOD of 3.5 nM for Pb^2+^ in organic solvents, yet Sn^2+^ oxidation limits their long-term stability.^75^ These trade-offs make lead-free PQDs less competitive for ultra-sensitive applications but valuable for sustainable contexts.

Single-ion detection systems, such as CH_3_NH_3_PbBr_3_ for Hg^2+^ (ref. 59) and CsSnX_3_ for Pb^2+^,^75^ provide high selectivity for specific heavy metal ions, making them suitable for targeted applications like lubricant quality control. In contrast, multi-ion sensors, such as Eu^3+^-doped Cs_3_Bi_2_Cl_6_/Cs_3_Bi_2_Cl_9_ for Cu^2+^ and Fe^3+^ (LODs of 6.23 μM and 3.6 μM)^80^ or MASnBr_3_ for Fe^3+^ and Cr^6+^,^68^ offer broader applicability but often at the expense of higher LODs. Ratiometric designs, like Mn^2+^-doped CsPbCl_3_ for Cu^2+^ (LOD 22.12 nM),^69^ improve specificity in complex matrices through dual-emission signals. Lead-based PQDs are preferred for applications demanding ultra-high sensitivity and rapid response, such as industrial solvent monitoring, but require advanced stabilization to overcome toxicity and aqueous instability. Lead-free PQDs excel in eco-friendly applications, particularly in aqueous environments like wastewater treatment, despite their lower sensitivity. Single-ion sensors offer high specificity, while multi-ion and ratiometric sensors provide versatility for complex systems. Future advancements should focus on enhancing the PLQY of lead-free PQDs, improving stability through novel ligands, and integrating computational tools to optimize sensor design for industrial scalability (Table 4).

**Table 4:** Comparative analysis of lead-based and lead-free PQD sensors for heavy metal ion detection

PQD type	Composition	Target ion	LOD	Selectivity	Stability	Application environment	Industrial applications	Advantages	Disadvantages	Ref.
Lead-based	CH_3_NH_3_PbBr_3_	Hg^2+^	0.124 nM	High (ionic radius similarity with Pb^2+^)	Moderate (moisture-sensitive)	Organic solvents (toluene)	Environmental monitoring	High PLQY (50.28%), rapid response (<10 s)	Lead toxicity, limited aqueous stability	59
Lead-based	CsPbX_3_	Cu^2+^	2 nM to 2 μM	Moderate (sensitive to transition metals)	Moderate (ligand-dependent)	Organic solvents (hexane)	Lubricants	Tunable bandgap, high sensitivity	Lead toxicity, poor aqueous stability	76
Lead-based	CsPbBr_3_	Cu^2+^	0.1 nM	High (oleylamine enhances coordination)	Moderate (stable in hexane)	Organic solvents (hexane)	Industrial solvents	Ultra-low LOD, fast response (<10 s)	Limited to non-aqueous media	62
Lead-based	CsPbBr_3_	Cu^2+^	Not specified	High (oleylamine enables phase transfer)	Improved in aqueous media	Aqueous solutions	Water quality monitoring	Aqueous compatibility	Lead toxicity, complex synthesis	61
Lead-based	CsPbBr_3_ with Au nanoclusters	Cu^2+^	Not specified	High (ratiometric design)	High (SiO_2_ encapsulation)	Aqueous solutions	Environmental samples	Visual color change, high stability	Complex nanocomposite fabrication	64
Lead-based	CsPbX_3_ in Zn-MOF	Cu^2+^	Not specified	High (MOF enhances selectivity)	High (MOF encapsulation)	Aqueous solutions	Industrial wastewater	Enhanced stability, high PLQY	Scalability challenges for MOFs	81
Lead-based	CsPbBr_3_ in PCN-333(Fe) MOF	Cu^2+^	1.63 nM	High (MOF-mediated coordination)	High (6.5-fold PL enhancement)	Aqueous solutions	Environmental monitoring	Ultra-low LOD, enhanced PL	Lead toxicity, MOF synthesis complexity	78
Lead-based	CsPbBr_3_@BBA	Cu^2+^	0.8 μM	Moderate (ligand-mediated)	High (water-dispersible)	Aqueous solutions	Food safety	Water compatibility, high stability	Moderate LOD, lead toxicity	77
Lead-based	CsPbBr_3_ in ZIF-8 MOF	Cu^2+^	2.64 nM	High (MOF enhances specificity)	High (15 day stability in water)	Aqueous solutions	Water quality monitoring	Ultra-low LOD, long-term stability	Lead toxicity, complex MOF integration	79
Lead-based	CsPbBr_3_–CsPb_2_Br_5_ with APTES	Fe^3+^	10 μM	High (APTES reduces non-specific interactions)	High (APTES passivation)	Aqueous solutions	Industrial effluents	Fast response (8 s), high stability	Moderate LOD, lead toxicity	67
Lead-based	CsPbBr_3_–CsPb_2_Br_5_ with ammonia hydroxide	Cd^2+^	1 μM	Moderate (ammonia hydroxide enhances accessibility)	Moderate (aqueous compatibility)	Aqueous solutions	Industrial effluents	Improved aqueous detection	Moderate LOD, lead toxicity	72
Lead-free	Cs_3_Bi_2_Br_9_:Eu^3+^	Cu^2+^	98.3 nM	High (Eu^3+^ doping enhances specificity)	High (stable in seawater)	Aqueous solutions (seawater)	Marine monitoring	Lead-free, high photostability	Lower PLQY (42.4%) than lead-based	60
Lead-free	MASnBr_3_ with PEI	Fe^3+^, Cr^6+^	Nanomolar range	High (PEI chelation enhances selectivity)	Moderate (ligand-dependent)	Aqueous and organic solutions	Environmental monitoring	Lead-free, multi-ion detection	Limited stability in harsh conditions	68
Lead-free	Cs_3_Bi_2_Cl_9_ with hydroxypropyl chitosan	Cr^6+^	0.27 μM	High (chitosan enhances ion accessibility)	High (aqueous compatibility)	Aqueous solutions (wastewater)	Wastewater treatment	Lead-free, high sensitivity	Moderate LOD compared to lead-based	71
Lead-free	Cs_3_Bi_2_Cl_6_/Cs_3_Bi_2_Cl_9_:Eu^3+^	Cu^2+^, Fe^3+^	6.23 μM (Cu^2+^), 3.6 μM (Fe^3+^)	Moderate (Eu^3+^ doping aids selectivity)	High (stable crystal structures)	Aqueous solutions	Environmental monitoring	Lead-free, multi-ion detection	Higher LOD than lead-based	80

### 4.4. Strategies for enhancing sensor performance

Several strategies have been developed to optimize the performance of PQD-based sensors, addressing limitations in stability, sensitivity, and selectivity for heavy metal detection.

#### 4.4.1. Surface passivation and ligand engineering

Surface passivation with tailored ligands enhances stability and selectivity. Hydroxypropyl chitosan-passivated Cs_3_Bi_2_Cl_9_ PQDs improve selectivity for Cr^6+^ by facilitating ion coordination, achieving an LOD of 0.27 μM in aqueous media.^71^ APTES-coated CsPbBr_3_–CsPb_2_Br_5_ PQDs enhance Fe^3+^ detection by reducing non-specific interactions, with an LOD of 10^−5^ M and a response time of 8 s.^67^ Poly(ethylenimine) ligands in MASnBr_3_ PQDs introduce chelating groups, improving Fe^3+^ selectivity through stable complex formation, critical for industrial fluid analysis.^68^ These ligand designs optimize ion accessibility while maintaining PQD stability.

#### 4.4.2. Encapsulation in stable matrices

Encapsulation in robust matrices, such as metal–organic frameworks (MOFs) or silica, protects PQDs from environmental degradation while maintaining analyte accessibility. CsPbBr_3_ PQDs encapsulated in ZIF-8 MOFs achieve an LOD of 2.64 nM for Cu^2+^ and retain stability in water for 15 days, suitable for industrial wastewater monitoring.^79^ PCN-333(Fe) MOF encapsulation enhances CsPbBr_3_ stability, achieving an LOD of 1.63 nM for Cu^2+^ with a 6.5-fold PL enhancement.^78^ Silica-encapsulated CsPbBr_3_@SiO_2_ systems support ratiometric Cu^2+^ detection with high stability in aqueous environments, ideal for industrial applications.^64^ These matrices balance protection and sensitivity, enabling robust sensor performance.^81^

#### 4.4.3. Doping with metal ions

Doping with metal ions like Mn^2+^ or Eu^3+^ enhances PL stability and enables advanced sensing modalities. Mn^2+^-doped CsPbCl_3_ PQDs exhibit dual emission for ratiometric Cu^2+^ detection, achieving an LOD of 22.12 nM with a PLQY of 52.48%.^19^ Eu^3+^-doped Cs_3_Bi_2_Br_9_ PQDs detect Cu^2+^ and Fe^3+^ with LODs of 6.23 μM and 3.6 μM, respectively, benefiting from improved photostability and polychromatic emission.^80^ Doping introduces additional emission bands, enhancing detection accuracy in industrial settings.^66^

#### 4.4.4. Ratiometric and visual sensing approaches

Ratiometric sensing improves accuracy by using dual-emission signals to mitigate environmental noise. CsPbBr_3_ PQDs combined with Au nanoclusters enable ratiometric Cu^2+^ detection, with visual color changes enhancing usability in industrial quality control.^64^ Cs_3_Bi_2_Cl_9_ PQDs facilitate visual detection of Cr^6+^ at 0.27 μM through distinct PL changes, suitable for on-site industrial monitoring.^71^

### 4.5. Industrial applications of PQD-based nanosensors for heavy metal detection

PQD-based nanosensors have demonstrated significant potential in industrial settings for detecting heavy metal ions in various matrices, ensuring quality control and preventing contamination. These applications are particularly relevant in industries such as chemical processing, manufacturing, and waste management, where heavy metal pollutants pose significant risks to product integrity and environmental safety. CsSnX_3_ PQDs are highly effective for detecting Pb^2+^ in lubricants and organic solutions, achieving an LOD of 3.5 nM through optimized synthesis methods that enhance PL stability.^75^ Their design leverages the high compatibility of lead-free PQDs with non-aqueous media, making them ideal for monitoring industrial fluids used in machinery and automotive applications. The rapid response time (<15 s) and high selectivity over competing ions like Zn^2+^ or Ca^2+^ ensure reliable detection of trace Pb^2+^, which is critical for maintaining lubricant quality and preventing equipment corrosion.^75^ The robustness of CsSnX_3_ PQDs in organic solvents, combined with their eco-friendly composition, positions them as a sustainable solution for industrial monitoring.

CsPbX_3_ PQDs are employed to detect Cu^2+^ in organic solvents used in chemical processing, achieving a detection range of 2 × 10^−9^ to 2 × 10^−6^ M. Their high PLQY (up to 90%) and sensitivity make them suitable for monitoring trace Cu^2+^ in solvents used for catalysis or material synthesis, where even low concentrations can affect product quality.^76^ For instance, Cu^2+^ contamination in organic solvents can catalyze unwanted side reactions, and PQD-based nanosensors provide a rapid and precise method to ensure solvent purity. The use of OAm ligands in these systems enhances selectivity by facilitating Cu^2+^ coordination, minimizing interference from other metal ions.^62^ In industrial wastewater treatment, Cs_3_Bi_2_Br_9_ PQDs are utilized to detect Cu^2+^ with an LOD of 98.3 nM, leveraging their high photostability and resistance to aqueous degradation.^60^ These lead-free PQDs are particularly valuable in monitoring effluent streams from metal plating or electronics manufacturing, where Cu^2+^ contamination is a common concern. Their ability to function in aqueous environments without significant PL degradation makes them suitable for continuous monitoring systems, ensuring compliance with environmental regulations.^60^ Additionally, hydroxypropyl chitosan-passivated Cs_3_Bi_2_Cl_9_ PQDs detect Cr^6+^ in wastewater with an LOD of 0.27 μM, offering a visual detection method that is practical for on-site industrial applications.^71^ The distinct PL changes induced by Cr^6+^ enable rapid identification of contamination, facilitating timely remediation.

CsPbBr_3_–CsPb_2_Br_5_ PQDs, modified with ammonia hydroxide, are applied to detect Cd^2+^ in industrial effluents, achieving an LOD of 10^−6^ M. These sensors are designed for wastewater from industries like battery manufacturing, where Cd^2+^ is a prevalent pollutant. The ammonia hydroxide modification enhances surface accessibility, improving sensitivity in aqueous media, which is critical for real-time monitoring of industrial discharge.^72^ Similarly, APTES-coated CsPbBr_3_–CsPb_2_Br_5_ PQDs detect Fe^3+^ with an LOD of 10^−5^ M, suitable for monitoring iron contamination in industrial fluids used in steel production or chemical synthesis.^67^ The high stability of APTES coatings ensures reliable performance under harsh industrial conditions. Ratiometric sensing systems, such as CsPbBr_3_ PQDs paired with Au nanoclusters, are employed for Cu^2+^ detection in industrial solvents, providing visual color changes that enhance usability for on-site quality control.^64^ These systems are particularly valuable in industries requiring rapid, non-invasive detection methods, such as petrochemical processing, where Cu^2+^ contamination can degrade product performance. The dual-emission signals improve accuracy by mitigating environmental noise, ensuring precise quantification.^64^

## 5. Comparative analysis with other sensing materials for heavy metal ion detection

This section evaluates PQDs against alternative sensing materials, including carbon quantum dots (CQDs), graphene quantum dots (GQDs), traditional semiconductor quantum dots (*e.g.*, CdS, CdTe, ZnS-based), and metal–organic frameworks (MOFs), for detecting heavy metal ions. The comparison emphasizes performance metrics such as limit of detection (LOD), selectivity, PLQY, synthesis complexity, and applicability in environmental and industrial contexts. By benchmarking PQDs against these materials, their unique strengths, limitations, and synergistic potential are highlighted, ensuring a distinct perspective from prior mechanistic, design, and application discussions.

### 5.1. Comparative analysis of PQDs and nanomaterials for heavy metal sensing

Detecting heavy metal ions in environmental, industrial, and biological matrices requires materials with high sensitivity, selectivity, and practical applicability. PQDs, CQDs, GQDs, semiconductor quantum dots, and MOFs each offer distinct properties for this purpose. CQDs, derived from carbon-based precursors, feature surface functional groups (*e.g.*, NH_2_, COOH) that enhance metal ion interactions, making them eco-friendly and suitable for aqueous environments.^82,83^ GQDs, a subset of CQDs, leverage surface plasmon resonance (SPR) for detection, offering high stability in water.^84^ Semiconductor QDs like CdS and CdTe provide tunable optical properties but are limited by toxicity.^85,86^ MOFs, often paired with luminescent nanomaterials, enhance stability and selectivity through their porous structures.^78,87^Table 5 provides a comprehensive comparison of these materials' performance metrics, including LOD and PLQY, across various detection environments.

**Table 5:** Comparative performance of PQDs and other sensing materials for heavy metal ion detection

Material	Target ion	LOD	Selectivity	PLQY (%)	Synthesis complexity	Application environment	Ref.
CH_3_NH_3_PbBr_3_ PQD	Hg^2+^	0.124 nM	High (cation exchange)	50.28	Moderate (LARP)	Organic solvents (toluene)	59
CH_3_NH_3_PbBr_3_@MOF-5 PQD	Cu^2+^	0.5 nM	High (MOF-mediated coordination)	80	High (MOF encapsulation)	Aqueous solutions	87
CsPbBr_3_/PMMA FM	Cu^2+^	10^−15^ M	High (FRET-based)	88	High (electrospinning)	Aqueous/organic (ethanol solution)	88
CsPbX_3_ PQD	Cu^2+^, Yb^3+^	2 nM	Moderate (transition metal sensitivity)	79	Moderate (hot-injection)	Organic solvents (hexane)	76
CsPbBr_3_ PQD	Cu^2+^	0.1 nM	High (oleylamine coordination)	81	Moderate (hot-injection)	Organic solvents (hexane)	62
CsPbBr_3_ PQD (OAm phase transfer)	Cu^2+^	0.5 nM	High (phase transfer enhances access)	80	High (phase transfer)	Aqueous solutions	61
Cs_3_Bi_2_Br_9_:Eu^3+^ PQD	Cu^2+^	98.3 nM	High (doping specificity)	42.4	Moderate (hydrothermal)	Aqueous (seawater)	89
MASnBr_3_ PQD (PEI-capped)	Fe^3+^, Cr^6+^	1 nM	High (PEI chelation)	14.6	Moderate (LARP)	Aqueous/organic solutions	68
CsPbCl_3_:Mn^2+^ PQD	Cu^2+^	22.12 nM	High (ratiometric dual emission)	52.48	Moderate (hot-injection)	Aqueous solutions	69
Cs_3_Bi_2_Cl_9_ PQD (chitosan-passivated)	Cr^6+^	0.27 μM	High (ligand-enhanced coordination)	35	Moderate (hydrothermal)	Aqueous (wastewater)	71
Cs_3_Bi_2_Cl_6_/Cs_3_Bi_2_Cl_9_:Eu^3+^ PQD	Cu^2+^, Fe^3+^	6.23 μM (Cu^2+^), 3.6 μM (Fe^3+^)	Moderate (doping aids selectivity)	35	Moderate (hydrothermal)	Aqueous solutions	80
CsSnX_3_ PQD	Pb^2+^	3.5 nM	High (ligand-mediated specificity)	25	Moderate (hot-injection)	Organic solvents (lubricants)	75
CsPbBr_3_@PCN-333(Fe) MOF PQD	Cu^2+^	1.63 nM	High (MOF-mediated coordination)	85	High (MOF encapsulation)	Aqueous solutions	78
CsPbBr_3_@ZIF-8 MOF PQD	Cu^2+^	2.64 nM	High (MOF enhances specificity)	84	High (MOF encapsulation)	Aqueous solutions	79
CsPbBr_3_–CsPb_2_Br_5_ PQD (APTES)	Fe^3+^	10 μM	High (APTES reduces non-specificity)	85	Moderate (hot-injection)	Aqueous (industrial effluents)	67
CsPbBr_3_–CsPb_2_Br_5_ PQD (NH_4_OH)	Cd^2+^	1 μM	Moderate (NH_4_OH enhances access)	83	Moderate (hot-injection)	Aqueous (industrial effluents)	72
Carbon quantum dots (CQD thin film)	Pb^2+^, Ni^2+^, Mn^2+^, Co^2+^, Cr^3+^	6 nM	Moderate (fluorescence quenching)	20	High (thin film deposition)	Aqueous (real water samples)	82
C_3_B_2_ quantum dots	Cd^2+^, Hg^2+^, Ni^2+^, As^3+^, Pb^2+^	10 nM	High (>80% sensitivity, adsorption)	30	High (DFT-based design)	Aqueous (wastewater)	90
S@PSCA CQDs	Pb^2+^, Ag^+^	3.83 nM (Pb^2+^), 9 μM (Ag^+^)	Moderate (colorimetric changes)	20	Moderate (hydrothermal)	Aqueous solutions	91
SO4@PSCA CQDs	Ag^+^	8.2 nM	Moderate (colorimetric changes)	20	Moderate (hydrothermal)	Aqueous solutions	91
SnO_2_ quantum dots	Ni^2+^	10 nM	Moderate (Sn vacancy interactions)	25	Moderate (hydrolysis)	Aqueous (deionized, reclaimed, sea)	92
Nitrogen-doped CQDs (N-CQDs)	Fe^3+^, Hg^2+^	35.8 nM (Fe^3+^), 6.8 nM (Hg^2+^)	High (fluorescence quenching)	20	High (oxidation)	Aqueous (pond, sea, well)	93
CQDs (blue crab shells)	Pb^2+^, Hg^2+^	5 nM	High (NH_2_, COOH, OH groups)	50	Moderate (microwave)	Aqueous (aquaculture)	83
Graphene QDs (S,N-GQDs)	Hg^2+^	16.32 nM	High (SPR-based)	30	Moderate (pyrolysis)	Aqueous (tap, river)	84
CdS QDs (DMSO-capped)	Cu^2+^, Hg^2+^, Pb^2+^	179.5 nM (Cu^2+^), 58 μM (Hg^2+^), 60 μM (Pb^2+^)	High (colorimetric)	30	Moderate (chemical synthesis)	Aqueous (polluted samples)	85
CdTe QDs (TGA, GSH, L-cyst.)	Cr^3+^, Pb^2+^	100 nM	High (turn-on for Cr^3+^, Pb^2+^)	58	Moderate (aqueous phase)	Aqueous/organic solutions	94
CdTe QDs (TGA-capped)	Cu^2+^	50 nM	High (1 : 1 complexation)	60	Moderate (one-step aqueous)	Aqueous solutions	95
CdTe/CdS/ZnS QDs	Hg^2+^	16.32 nM	High (fluorescence quenching)	59	High (multi-step aqueous)	Aqueous (tap, river, agricultural)	96
CdSe/ZnS QDs (TGA-capped)	Cr^3+^, Cu^2+^	100 nM	High (turn-off fluorescence)	60	Moderate (aqueous phase)	Aqueous solutions	97
CdS QDs (GSH-capped)	Cu^2+^, Hg^2+^, Mg^2+^	100 nM	Moderate (fluorescence quenching)	59	Moderate (one-pot)	Foods (aqueous media)	98
CuInS2/ZnS QDs (GSH-capped)	Cu^2+^	63 nM	High (aggregation-based quenching)	55	Moderate (aqueous)	Aqueous solutions	99
Co/Ag@ZnS QDs	Hg^2+^	130 nM	High (fluorescence quenching)	27.6	Moderate (co-precipitation)	Aqueous (real water samples)	100
g-C_3_N_4_/CeO_2_ heterostructure	Cu^2+^, Hg^2+^, Ag^+^	26.2 μM, 17.5 μM, 14.9 μM	Moderate (binding affinity)	10	High (co-precipitation)	Aqueous solutions	101
Starch-capped CdS QD	Hg^2+^, Cu^2+^	100 nM	High (cation exchange reaction)	30	Moderate (aqueous)	Aqueous solutions	86

CQDs, synthesized *via* hydrothermal or microwave methods, typically exhibit PLQY of 20–50% and LODs of 5–35.8 nM for ions like Pb^2+^ and Hg^2+^ in water samples.^82,93^ Their broad emission spectra limit sensitivity compared to PQDs. GQDs, with similar PLQY (20–30%), achieve LODs of 16.32 nM for Hg^2+^ using SPR, but their pyrolysis-based synthesis is less scalable.^84^ Semiconductor quantum dots, such as CdTe, offer higher PLQY (50–60%) and LODs of 50–179.5 nM, but cadmium toxicity and complex synthesis hinder their use.^85,98^ MOFs, while not inherently luminescent, enhance the performance of embedded fluorophores, though their fabrication is intricate.^78,87^ PQDs combine high PLQY, narrow emission, and moderate synthesis complexity, making them versatile for both organic and aqueous applications. The choice of material depends on the application context. CQDs and GQDs excel in eco-friendly aqueous detection, while semiconductor QDs suit high-sensitivity needs despite toxicity concerns. MOFs enhance stability but require complex integration. PQDs offer a balanced profile of sensitivity, tunability, and adaptability, positioning them as a leading platform for heavy metal detection across diverse matrices.

### 5.2. Advantages of PQDs for heavy metal ion detection

PQDs, particularly lead-based variants like CsPbX_3_ and CH_3_NH_3_PbX_3_, exhibit exceptional optical properties, including high PLQY and narrow emission spectra, enabling ultra-low LODs (*e.g.*, 0.124 nM for Hg^2+^ with CH_3_NH_3_PbBr_3_).^59^ This optical precision supports ratiometric sensing, as seen in Mn^2+^-doped CsPbCl_3_ (LOD 22.12 nM for Cu^2+^), enhancing accuracy in complex matrices.^69^ Their tunable bandgap, adjusted *via* size or halide composition, allows tailored detection, such as CsPbBr_3_ achieving an LOD of 0.1 nM for Cu^2+^ in hexane through OAm coordination.^62^ Lead-free PQDs, like Cs_3_Bi_2_Br_9_:Eu^3+^, offer comparable performance (LOD 98.3 nM for Cu^2+^) with reduced toxicity, ideal for aqueous environments like seawater.^89^ Their rapid response times (<10 s) stem from efficient quenching mechanisms, such as cation exchange for Hg^2+^ detection with CH_3_NH_3_PbBr_3_, outperforming CQDs' slower responses.^59,82^ Integration into MOFs, as with CsPbBr_3_@PCN-333(Fe) (LOD 1.63 nM for Cu^2+^), enhances aqueous stability, making PQDs suitable for industrial wastewater monitoring.^78^Table 5 highlights these advantages, showing PQDs' superior LODs and versatility compared to other materials. PQDs' compatibility with organic solvents (*e.g.*, hexane, lubricants) and aqueous media *via* phase-transfer techniques (*e.g.*, oleylamine-modified CsPbBr_3_, LOD 0.5 nM for Cu^2+^) broadens their industrial applicability.^61^ In contrast, GQDs are limited to aqueous systems, and CdS QDs struggle with selectivity in complex matrices.^84,98^ The combination of high PLQY, tunability, and rapid response positions PQDs as a leading choice for high-performance heavy metal detection.

### 5.3. Limitations of alternative sensing materials

CQDs, despite their eco-friendliness, are constrained by lower PLQY (20–50%) and broader emission spectra, reducing sensitivity. For example, CQDs from blue crab shells achieve an LOD of 5 nM for Pb^2+^, but their PLQY (50%) is lower than CsPbBr_3_'s (81%).^62,83^ Their need for complex surface functionalization to enhance selectivity increases synthesis complexity.^91,93^ GQDs, with PLQY of 20–30%, achieve an LOD of 16.32 nM for Hg^2+^, significantly higher than PQDs' 0.124 nM, and their pyrolysis synthesis is less scalable.^59,84^ Semiconductor QDs like CdS and CdTe offer PLQY of 50–60% and LODs of 50–179.5 nM, but cadmium toxicity limits their environmental applications.^94–98^ Their multi-step synthesis is more complex than PQD hot-injection or LARP methods, and selectivity often requires multiple capping agents.^94,97^ MOFs, while enhancing stability when paired with PQDs, lack intrinsic luminescence and require intricate fabrication, limiting standalone use.^78,79^Table 5 underscores these limitations, showing higher LODs and synthesis challenges for alternative materials compared to PQDs' superior performance.

### 5.4. Synergistic approaches combining PQDs with other nanomaterials

Combining PQDs with MOFs, CQDs, or metal nanoclusters creates synergistic systems that enhance detection performance. Encapsulation in MOFs, such as CsPbBr_3_@PCN-333(Fe) (LOD 1.63 nM for Cu^2+^) or CsPbBr_3_@ZIF-8 (LOD 2.64 nM), improves aqueous stability and analyte accessibility, ideal for wastewater monitoring.^78,79^ These composites leverage MOF porosity and PQD optical properties, overcoming the aqueous instability of lead-based PQDs. Hybrid systems with CQDs or Au nanoclusters enable ratiometric sensing, as seen with CsPbBr_3_ and Au nanoclusters for visual Cu^2+^ detection *via* FRET, enhancing accuracy in industrial solvents.^64^ CsPbBr_3_/PMMA fiber membranes achieve an LOD of 10^−15^ M for Cu^2+^, combining PQD fluorescence with polymer matrix stability.^88^ Doping with Eu^3+^ or Mn^2+^, as in Cs_3_Bi_2_Br_9_:Eu^3+^ (LOD 98.3 nM for Cu^2+^), enhances PLQY and selectivity in aqueous settings.^69,89^ These synergistic approaches address PQD limitations, offering performance superior to standalone CQDs or semiconductor quantum dots.

**Fig. 10:**
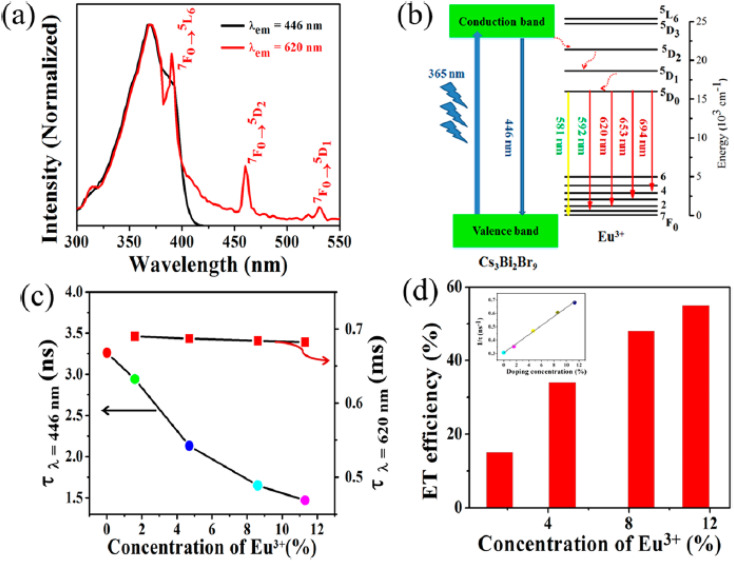
(a) Excitation spectra of Eu^3+^ (8.6 mol%)-doped Cs_3_Bi_2_Br_9_:PQDs at 446 and 620 nm. (b) Energy level diagram of Eu^3+^-doped Cs_3_Bi_2_Br_9_:PQDs. (c) Time-resolved PL decay at 446 nm and 620 nm (^5^D_0_ → ^7^F_j_) for undoped and Eu^3+^ (1.6–11.3 mol%)-doped samples. (d) Energy transfer efficiency from Cs_3_Bi_2_Br_9_:PQDs to Eu^3+^*vs.* concentration (inset: 446 nm decay constants). Reprinted with permission from ref. 89. Copyright 2019, American Chemical Society.

The provided figure illustrates key optical and energetic properties of Eu^3+^-doped Cs_3_Bi_2_Br_9_ PQDs combined with PeQD. Panel (a) presents the excitation spectra monitored at 446 nm and 620 nm, revealing distinct peaks at 365 nm and broader emissions influenced by Eu^3+^ doping, with emission wavelengths at 446 nm and 620 nm corresponding to transitions from the conduction band to the valence band and specific Eu^3+^ energy levels (^5^D_0_ → ^7^F_j_). This highlights the enhanced PL properties due to Eu^3+^ incorporation, which boosts PLQY and selectivity, as noted for Cs_3_Bi_2_Br_9_:Eu^3+^ with an LOD of 98.3 nM for Cu^2+^. Panel (b) depicts the energy level diagram, showing the alignment of Eu^3+^ levels (^5^D_0_ to ^5^D_4_ and ^7^F_0_ to ^7^F_6_) with the conduction and valence bands of Cs_3_Bi_2_Br_9_, facilitating efficient ET and FRET mechanisms that underpin the improved detection performance in hybrid systems. Panels (c) and (d) further elucidate the concentration-dependent behavior of Eu^3+^ doping. Panel (c) shows the decay time (*τ*) at 446 nm decreasing from 3.5 ns to 1.5 ns with increasing Eu^3+^ concentration (0–12%), indicating enhanced non-radiative energy transfer to Eu^3+^ ions, as supported by the inset's reverse exciton emission decay constants. Panel (d) quantifies the ET efficiency (*η*_ET_) and coefficient (*α*), peaking at 0.7 and 0.6, respectively, at 12% doping, alongside a bar graph of *η*_ET_, which underscores the optimal doping level for maximizing PLQY and sensing accuracy in aqueous environments. These findings reinforce the synergistic potential of Eu^3+^-doped PQDs with nanomaterials like MOFs or CQDs, enhancing stability and detection limits as demonstrated in systems like CsPbBr_3_@PCN-333(Fe) and CsPbBr_3_/PMMA fiber membranes (Fig. 10).

### 5.5. Benchmarking PQD performance against established sensors

PQDs consistently outperform alternative sensors in key metrics. For Hg^2+^, CH_3_NH_3_PbBr_3_ achieves an LOD of 0.124 nM, surpassing CdTe/CdS/ZnS (16.32 nM) and GQDs (16.32 nM) due to efficient cation exchange.^84,96^ For Cu^2+^, CsPbBr_3_ reaches an LOD of 0.1 nM, compared to CdS (179.5 nM) and CuInS_2_/ZnS (63 nM), leveraging rapid quenching mechanisms.^85,99^ Lead-free Cs_3_Bi_2_Cl_9_ detects Cr^6+^ at 0.27 μM, competitive with CdTe (100 nM), with added eco-friendliness.^71,94^ PQDs' selectivity, enhanced by tailored ligands and doping, matches or exceeds that of CdTe and CdS, which require complex functionalization.^62,94^ Their moderate synthesis complexity (hot-injection, LARP) contrasts with the multi-step processes of CdTe/CdS/ZnS or GQDs.^59,84^ PQDs' versatility across organic and aqueous environments, particularly when integrated with MOFs, positions them as a robust platform for industrial and environmental monitoring, outperforming alternative materials in sensitivity and applicability.

## 6. Challenges, future directions, and pathways to industrial adoption

PQDs have emerged as a transformative platform for heavy metal ion detection, offering exceptional sensitivity, tunable optical properties, and versatility across diverse matrices. Despite their promise, several challenges hinder their practical implementation and scalability, particularly in environmental, industrial, and biomedical applications. Addressing these obstacles is essential to fully harness the potential of PQDs for detecting heavy metal ions such as Hg^2+^, Cu^2+^, Cd^2+^, Fe^3+^, Cr^6+^, and Pb^2+^. This section delineates the primary challenges associated with PQD-based nanosensors and proposes future directions to overcome these limitations, paving the way for advanced, reliable, and commercially viable detection systems.

### 6.1. Challenges in PQD-based heavy metal ion detection

#### 6.1.1. Stability in aqueous and humid environments

The instability of PQDs, particularly lead-based variants like CsPbX_3_ and CH_3_NH_3_PbX_3_, in aqueous and humid environments remains a significant barrier. Exposure to moisture can degrade the perovskite lattice, leading to phase transitions or decomposition that diminish PLQY and sensing performance.^59,61^ For instance, CH_3_NH_3_PbBr_3_ PQDs exhibit reduced stability due to the volatility of organic cations, limiting their use in aqueous media such as wastewater or biological fluids.^59^ While encapsulation in matrices like metal–organic frameworks (MOFs) or silica enhances stability, as seen with CsPbBr_3_@ZIF-8 (stable for 15 days in water),^79^ these approaches increase synthesis complexity and may compromise analyte accessibility, reducing sensitivity for ions like Cu^2+^ or Cr^6+^.^78,79^ Lead-free PQDs, such as Cs_3_Bi_2_X_9_, offer improved moisture resistance but often at the cost of lower PLQY (20–50%), impacting detection limits.^60,71^ Achieving robust stability without sacrificing optical performance remains a critical challenge for practical applications in industrial effluents and environmental monitoring.

#### 6.1.2. Toxicity concerns of lead-based PQDs

The toxicity of lead-based PQDs, such as CsPbX_3_ and CH_3_NH_3_PbX_3_, poses significant environmental and health risks, particularly in applications involving water quality monitoring or biomedical diagnostics. The release of Pb^2+^ ions during degradation can contaminate the tested matrix, rendering these PQDs unsuitable for eco-sensitive applications.^59,62^ Although lead-free alternatives like Cs_3_Bi_2_X_9_ and CsSnX_3_ mitigate toxicity concerns, their lower PLQY and stability issues, such as Sn^2+^ oxidation in CsSnX_3_, limit their competitiveness.^68,75^ For example, Cs_3_Bi_2_Br_9_:Eu^3+^ achieves an LOD of 98.3 nM for Cu^2+^ in seawater, but its PLQY (42.4%) is significantly lower than that of CsPbBr_3_ (81%).^60,62^ Balancing toxicity reduction with high sensitivity and stability is a pressing challenge for widespread adoption.

#### 6.1.3. Interference from coexisting ions and complex matrices

Selectivity in complex matrices, such as industrial wastewater or biological fluids, is hindered by interference from coexisting ions (*e.g.*, Na^+^, K^+^, Ca^2+^) and organic species. While ligands like OAm or PE enhance selectivity for specific ions like Cu^2+^ or Fe^3+^, non-specific interactions can lead to false positives or reduced sensitivity.^62,68^ For instance, CsPbBr_3_ PQDs show high selectivity for Cu^2+^ in hexane but face challenges in aqueous environments with multiple ions, where LODs increase from 0.1 nM to 0.8 μM.^77^ Ratiometric designs, such as CsPbBr_3_ with Au nanoclusters, mitigate some interference through dual-emission signals, but their complexity limits scalability.^64^ Developing sensors that maintain high selectivity in real-world, multi-ion environments is a critical hurdle.

#### 6.1.4. Scalability and reproducibility of sensor fabrication

The scalability of PQD synthesis and sensor fabrication remains challenging due to batch-to-batch variations in size, crystallinity, and ligand passivation. Methods like hot-injection yield high-quality PQDs with PLQY > 80%, but their high-temperature, inert-atmosphere requirements limit large-scale production.^62^ Ligand-assisted reprecipitation (LARP) is more scalable but produces PQDs with broader size distributions and lower PLQY (50–70%), affecting reproducibility in sensing applications.^68^ For example, variations in ligand density can alter ion accessibility, impacting the LOD for Cu^2+^ detection.^61^ Additionally, integrating PQDs into stable matrices like MOFs or polymers increases fabrication complexity, posing challenges for cost-effective, industrial-scale production.^78,79^ Ensuring consistent sensor performance across large-scale manufacturing is essential for commercial applications.

#### 6.1.5. Lack of standardization for commercial and regulatory applications

The absence of standardized protocols for PQD-based sensor design, calibration, and validation hinders their regulatory approval and commercial adoption. Variations in synthesis methods, ligand types, and detection conditions lead to inconsistent performance metrics, complicating comparisons across studies. For instance, LODs for Cu^2+^ detection range from 0.1 nM (CsPbBr_3_ in hexane) to 6.23 μM (Cs_3_Bi_2_Cl_6_:Eu^3+^ in aqueous media), reflecting diverse testing environments and sensor designs.^62,80^ Regulatory frameworks for environmental and biomedical applications require standardized testing conditions and safety assessments, particularly for lead-based PQDs, which face scrutiny due to toxicity concerns.^59^ Establishing universal standards for PQD-based nanosensors is critical to ensure reliability and compliance in practical settings.

### 6.2. Future directions for PQD-based sensors

To overcome the aforementioned challenges and advance PQD-based nanosensors for heavy metal ion detection, innovative strategies and interdisciplinary approaches are needed. The following directions outline opportunities to enhance stability, sensitivity, selectivity, and scalability, aligning with the demands of industrial, environmental, and biomedical applications.

#### 6.2.1. Development of stable and eco-friendly lead-free PQDs

Advancing lead-free PQDs, such as Cs_3_Bi_2_X_9_, CsSnX_3_, or double perovskites (*e.g.*, Cs_2_AgInCl_6_), is crucial to address toxicity and stability concerns. Enhancing the PLQY of lead-free PQDs through doping with ions like Eu^3+^ or Mn^2+^ can improve sensitivity, as demonstrated by Eu^3+^-doped Cs_3_Bi_2_Br_9_ (LOD 98.3 nM for Cu^2+^).^60^ Novel synthesis methods, such as microwave-assisted or solvothermal approaches, can improve crystallinity and reduce defects, enhancing PLQY and stability in aqueous environments.^71^ Exploring new compositions, such as germanium-based or antimony-based perovskites, could yield environmentally benign PQDs with optical properties comparable to lead-based systems, enabling sustainable applications in wastewater and seawater monitoring.

#### 6.2.2. Advanced encapsulation and surface engineering

Innovative encapsulation strategies can enhance PQD stability without compromising analyte accessibility. Hybrid matrices combining MOFs with polymers or silica, such as CsPbBr_3_@PCN-333(Fe) (LOD 1.63 nM for Cu^2+^), offer robust protection and high sensitivity.^78^ Developing stimuli-responsive coatings that allow selective analyte penetration could further improve selectivity in complex matrices. Ligand engineering, such as using zwitterionic or multidentate ligands, can enhance water compatibility and ion-specific interactions, as seen with hydroxypropyl chitosan-passivated Cs_3_Bi_2_Cl_9_ for Cr^6+^ detection (LOD 0.27 μM).^71^ Machine learning-guided ligand design can optimize surface chemistry, predicting ligand–ion interactions to enhance selectivity and sensitivity for ions like Fe^3+^ or Cd^2+^.

#### 6.2.3. Integration with advanced detection platforms

Integrating PQD-based nanosensors with advanced platforms, such as microfluidics or smartphone-based systems, can enhance portability and real-time monitoring capabilities. Microfluidic devices can enable precise control over sample delivery, improving detection consistency in industrial effluents or biological fluids. For example, CsPbBr_3_–CsPb_2_Br_5_ PQDs integrated into microfluidic chips could detect Cd^2+^ with high reproducibility.^72^ Smartphone-based sensors, leveraging PQD fluorescence for visual or ratiometric detection, offer cost-effective solutions for on-site monitoring in industrial settings, as demonstrated by CsPbBr_3_–Au nanocluster systems for Cu^2+^.^64^ These platforms can democratize access to high-sensitivity detection, particularly in resource-limited environments.

#### 6.2.4. Machine learning and computational approaches

Machine learning (ML) and computational modeling, such as DFT and molecular dynamics (MD), can optimize PQD sensor design. ML algorithms can predict optimal PQD compositions and ligand configurations for specific ions, reducing experimental trial-and-error. For instance, DFT studies have elucidated Hg^2+^-induced mid-gap states in CH_3_NH_3_PbBr_3_, guiding sensor optimization.^59^ ML-driven analysis of quenching mechanisms can enhance selectivity by identifying ion-specific interactions in complex matrices, improving performance for multi-ion detection.^80^ Computational simulations can also model ion diffusion kinetics, informing ligand designs that balance stability and sensitivity, critical for detecting low-concentration ions like Pb^2+^ in lubricants.^75^

#### 6.2.5. Standardization and scalable fabrication

Establishing standardized protocols for PQD synthesis, sensor calibration, and performance evaluation is essential for commercial and regulatory acceptance. Developing scalable synthesis methods, such as continuous-flow hot-injection or automated LARP systems, can improve reproducibility and reduce costs. For example, microwave-assisted synthesis offers rapid, uniform nucleation, suitable for large-scale production of Cs_3_Bi_2_X_9_ PQDs.^71^ Standardizing performance metrics, such as LOD and linear range, across diverse matrices (*e.g.*, organic solvents, wastewater) will facilitate comparisons and regulatory approval. Collaborative efforts between researchers, industry, and regulatory bodies can establish guidelines for PQD-based sensors, ensuring safety and reliability in applications like industrial wastewater treatment.

#### 6.2.6. Multi-ion and multiplexed detection systems

Developing PQD-based nanosensors capable of detecting multiple heavy metal ions simultaneously will enhance their utility in complex industrial and environmental matrices. Multi-ion sensors, such as Eu^3+^-doped Cs_3_Bi_2_Cl_6_/Cs_3_Bi_2_Cl_9_ for Cu^2+^ and Fe^3+^ detection (LODs 6.23 μM and 3.6 μM), demonstrate the potential for multiplexed systems.^80^ Ratiometric designs with dual-emission PQDs, such as Mn^2+^-doped CsPbCl_3_, can improve accuracy by mitigating interference, ideal for industrial solvents or effluents.^69^ Combining PQDs with other nanomaterials, like CQDs or metal nanoclusters, can enable multiplexed detection with visual readouts, enhancing usability in real-time quality control.^64^ Future research should focus on optimizing these systems for simultaneous detection of ions like Hg^2+^, Cu^2+^, and Cr^6+^, addressing the needs of diverse applications.

#### 6.2.7. Eco-friendly and biocompatible sensor designs

To address regulatory and environmental concerns, future PQD-based nanosensors should prioritize eco-friendly and biocompatible designs. Lead-free PQDs with enhanced PLQY and stability, such as doped Cs_3_Bi_2_X_9_ or Cs_2_AgInCl_6_, can replace toxic lead-based systems in biomedical and environmental applications.^60^ Biocompatible ligands, such as peptides or polysaccharides, can improve safety for biological fluid analysis, enabling detection of heavy metals in clinical settings. For instance, integrating Cs_3_Bi_2_Cl_9_ with biocompatible coatings could enable Fe^3+^ detection in blood samples with minimal toxicity.^71^ These advancements will broaden the applicability of PQD-based sensors, aligning with global sustainability goals.

### 6.3. Commercialization pathways and industrial integration

The transition of perovskite quantum dot (PQD)-based nanosensors from laboratory research to industrial applications requires strategic pathways to ensure scalability, cost-effectiveness, and seamless integration into existing industrial workflows. While PQD sensors demonstrate exceptional sensitivity and selectivity for heavy metal ion detection, their commercial adoption hinges on addressing economic, regulatory, and operational challenges, distinct from their technical performance in specific applications.

#### 6.3.1. Cost-effective synthesis and manufacturing

Scaling up PQD synthesis for industrial use demands cost-effective methods that maintain high PLQY and reproducibility. Techniques like ligand-assisted reprecipitation (LARP) and continuous-flow hot-injection offer scalable alternatives to batch-based methods, reducing production costs while ensuring uniform PQD quality.^68^ For instance, automating LARP processes could lower the cost of producing lead-free PQDs like Cs_3_Bi_2_X_9_, making them economically viable for large-scale environmental monitoring.^60^ Collaborative partnerships with chemical manufacturing industries can optimize precursor sourcing and waste management, further reducing costs. Additionally, developing modular synthesis platforms that integrate quality control metrics, such as real-time PLQY monitoring, can enhance batch consistency, addressing reproducibility concerns for commercial production.

#### 6.3.2. Integration into industrial monitoring systems

Integrating PQD-based nanosensors into existing industrial systems, such as continuous monitoring platforms for wastewater treatment or quality control in chemical processing, requires compatibility with automated and IoT-enabled infrastructures. For example, embedding CsPbBr_3_@ZIF-8 sensors into inline water quality analyzers can enable real-time detection of Cu^2+^ in industrial effluents, with data transmitted to centralized control systems.^79^ Developing plug-and-play sensor modules that interface with standard industrial equipment, such as spectroscopy units or microfluidic systems, can streamline adoption. These modules should incorporate robust encapsulation (*e.g.*, MOFs or silica) to ensure stability under harsh industrial conditions, such as high temperatures or corrosive environments, without compromising analyte accessibility.^78^

#### 6.3.3. Regulatory compliance and market readiness

Achieving regulatory approval for PQD-based nanosensors involves addressing safety and environmental concerns, particularly for lead-based PQDs. Lead-free PQDs, such as Cs_3_Bi_2_Br_9_:Eu^3+^, align better with environmental regulations due to their reduced toxicity, making them more viable for markets with stringent standards, such as the European Union's REACH framework.^60^ Establishing standardized testing protocols for sensor performance, including LOD, selectivity, and stability under diverse conditions, is critical for regulatory acceptance. Collaborations with regulatory bodies can facilitate the development of certification pathways, ensuring PQD sensors meet industry standards for applications in wastewater treatment, chemical manufacturing, and environmental monitoring. Market readiness also requires demonstrating long-term reliability through field trials, such as deploying CsSnX_3_ sensors in lubricant quality control systems to validate performance over extended periods.^75^

#### 6.3.4. Economic viability and market differentiation

To compete with established sensing technologies, such as carbon QDs or semiconductor quantum dots, PQD-based nanosensors must offer clear economic advantages. Their high sensitivity (*e.g.*, LOD of 0.1 nM for Cu^2+^ with CsPbBr_3_) and versatility across organic and aqueous media provide a competitive edge.^62^ Positioning PQD sensors as cost-effective solutions for high-precision applications, such as detecting trace Pb^2+^ in lubricants, can attract industries seeking to minimize equipment downtime and contamination risks.^75^ Developing portable, user-friendly devices, such as smartphone-integrated PQD sensors for on-site Cu^2+^ detection, can target niche markets like small-scale chemical processing or environmental consulting, enhancing market penetration.^64^ Strategic partnerships with sensor manufacturers and distributors can accelerate market entry, leveraging existing supply chains to reduce time-to-market.

## 7. Conclusion

This comprehensive review underscores the transformative potential of PQDs as advanced platforms for heavy metal ion detection, marking the first systematic evaluation of their application in this domain. PQDs, with their high photoluminescence quantum yield, narrow emission spectra, and tunable optical properties, offer unparalleled sensitivity and selectivity for detecting ions such as Hg^2+^, Cu^2+^, Cd^2+^, Fe^3+^, Cr^6+^, and Pb^2+^. Their efficacy, driven by mechanisms like cation exchange and electron transfer, is enhanced by advanced synthesis methods and integration with stable matrices like metal–organic frameworks. Lead-based PQDs achieve sub-nanomolar detection limits, while lead-free variants address environmental concerns, broadening their applicability. The versatility of PQDs enables their use in industrial wastewater treatment and lubricant quality control, ensuring compliance with environmental regulations and product integrity. Despite these advancements, challenges such as aqueous instability, toxicity of lead-based PQDs, and scalability of synthesis methods remain. Future research should focus on developing stable, eco-friendly PQDs through novel encapsulation techniques and ligand designs, alongside computational modeling to optimize ion–PQD interactions. Comparative studies with other nanomaterials, such as carbon quantum dots, will further elucidate their unique advantages. By addressing these challenges, PQDs can transition from laboratory innovations to practical sensing solutions, offering rapid, cost-effective, and portable detection for environmental and industrial applications. This review lays a foundation for advancing PQD-based sensors, guiding researchers toward innovations that enhance sensitivity, selectivity, and scalability, ultimately contributing to sustainable monitoring of heavy metal ion contamination.

## Author contributions

Ahmad Mohebi and Suleiman Ibrahim Mohammad conceptualized the study. Asokan Vasudevan, I. B. Sapaev, and Munthar Kadhim Abosaoda developed the methodology. Chou-Yi Hsu, Malatesh Akkur, and Alok Kumar Mishra conducted the investigation. Gaganjot Kaur and Rajesh Singh handled data curation. Suleiman Ibrahim Mohammad and Ahmad Mohebi drafted the original manuscript, while Ahmad Mohebi, Asokan Vasudevan, and Chou-Yi Hsu reviewed and edited it. I. B. Sapaev and Munthar Kadhim Abosaoda contributed to visualization. Ahmad Mohebi supervised and managed the project. Suleiman Ibrahim Mohammad secured funding. All authors have read and agreed to the published version of the manuscript.

## Conflicts of interest

There are no conflicts to declare.

## Data Availability

No primary research results, software or code have been included and no new data were generated or analysed as part of this review.
